# Supplementary material for: pathVar: a new method for pathway-based interpretation of gene expression variability
Source: PeerJ. 2017 May 23;5:e3334. doi: 10.7717/peerj.3334 (PMC5444375; doi:10.7717/peerj.3334)
Supplement: Supplemental Information 1 [file peerj-05-3334-s001.docx]

**Supplementary Data File**

***Supplementary Figures***

**Figure S1. Hypothetical example of variability count distributions for a pathway and a reference.** **A.** The reference distribution corresponds to a hypothetical data set with 10,000 genes where 2,500 genes are in the low variability category, 5000 in the medium and 2500 in the high variability category. **B.** The count distribution of Pathway 1 contains a total of 100 genes from the dataset. From these 100 genes, 10 are in the low variability category, 55 in the medium, and 25 in the high variability category. The *pathVar* method assesses how different these two distributions are from each other using either an exact test or a Chi-squared test.


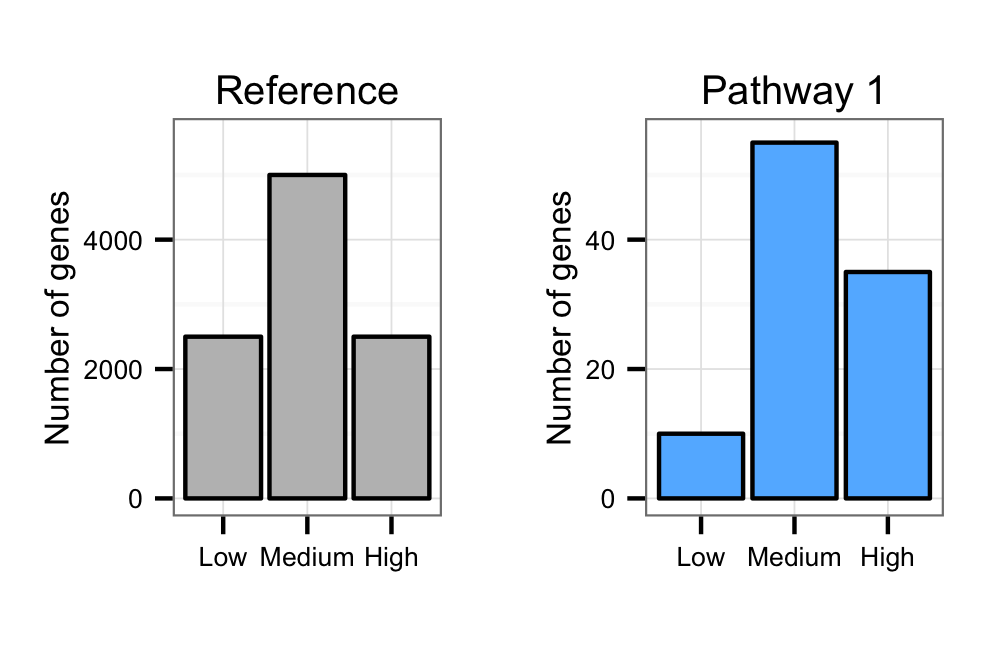


**Figure S2. Summarizing the main functional themes in significant REACTOME terms obtained for the one-group *pathVar* analysis of the human embryonic stem cell lines.** Classification of the statistically significant terms from REACTOME (adjusted P-value < 0.01) for **A**. the 125 terms for the Yan data set (passage 0), **B**. the 106 significant terms for the Yan data set (passage 10). **C**. the 69 significant terms for the Bock data set.

***Supplementary Tables***

**Table S1. Number of significant pathways obtained for the three stem cell data sets in the one-group case (adjusted P-value < 0.01).** Number of significant pathways obtained for **A.** KEGG and **B.** REACTOME in the one-group case when using *pathVar* based on either the variability statistic, or average gene expression. We also report the number of significant pathway terms that were identified in both the mean-based and variability-based analysis.

| Pathway Database | Datasets | Type | Genes | Samples | Significant for variability (SD) | Significant for mean | Intersection mean/variability |
| --- | --- | --- | --- | --- | --- | --- | --- |
| KEGG | Bock hESC | Microarray | 7632 | 20 | 25 | 24 | 9 |
|  | Bock iPSC | Microarray | 7646 | 12 | 19 | 21 | 9 |
|  | Yan hESC | Single cell RNA-seq | 6667 | 34 | 11 | 14 | 8 |
| REACTOME | Bock hESC | Microarray | 7632 | 20 | 65 | 138 | 43 |
|  | Bock iPSC | Microarray | 7646 | 12 | 66 | 149 | 42 |
|  | Yan hESC | Single cell RNA-seq | 6667 | 34 | 95 | 131 | 94 |

**Table S2. Statistically significant pathway terms from one-group *pathVar* analysis using the SD for the Bock human embryonic stem cell lines (adjusted P-value < 0.01). A.** KEGG pathways and **B.** REACTOME terms.

**A.** KEGG.


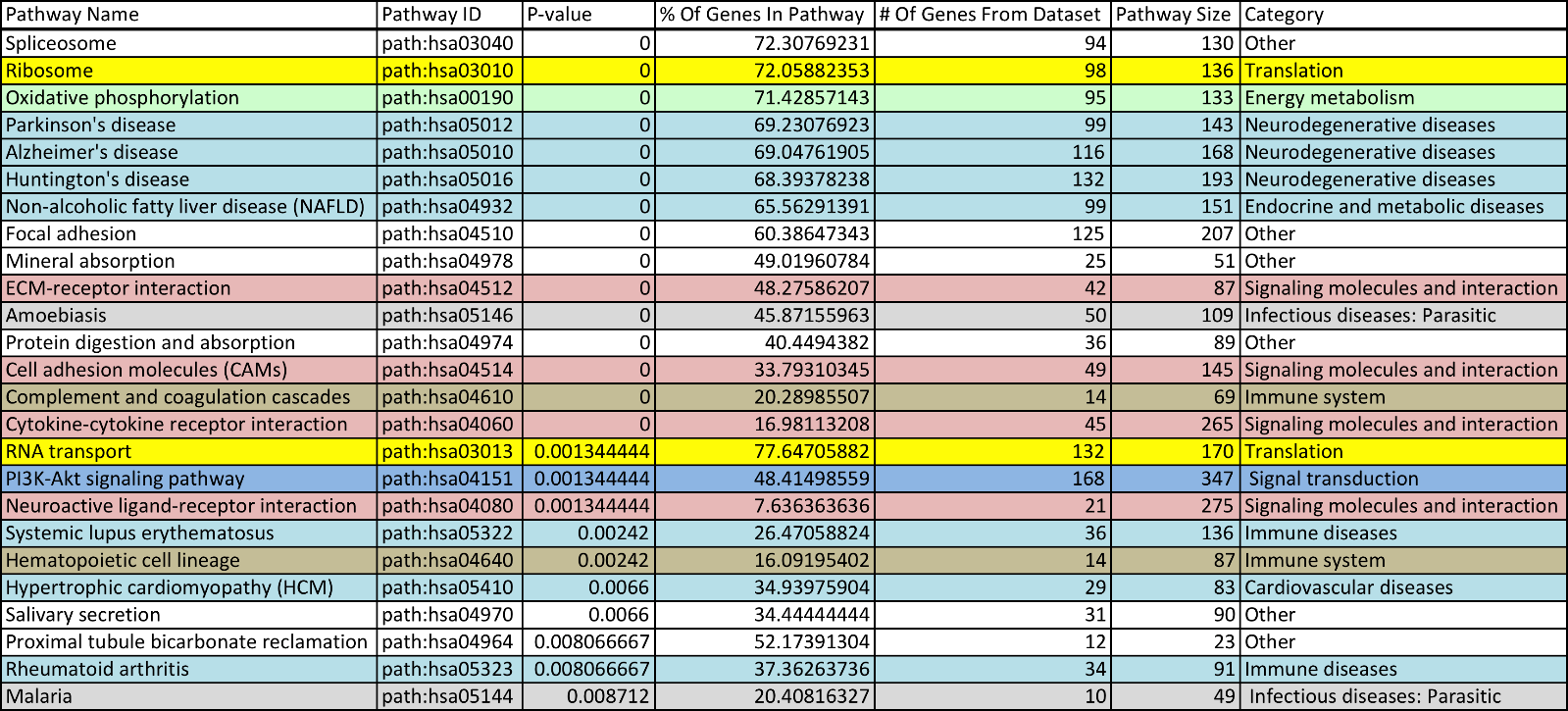


**B.** REACTOME.


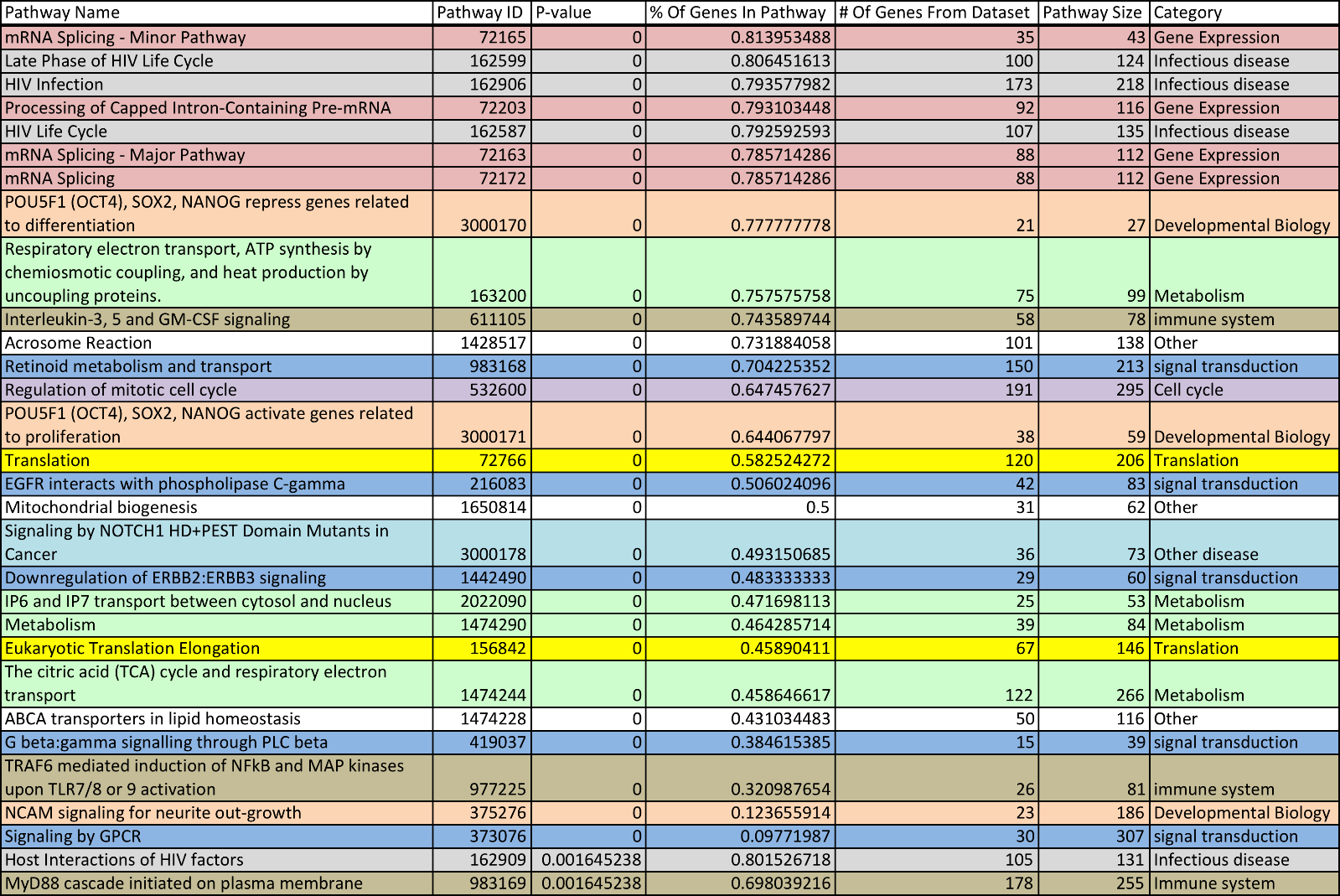


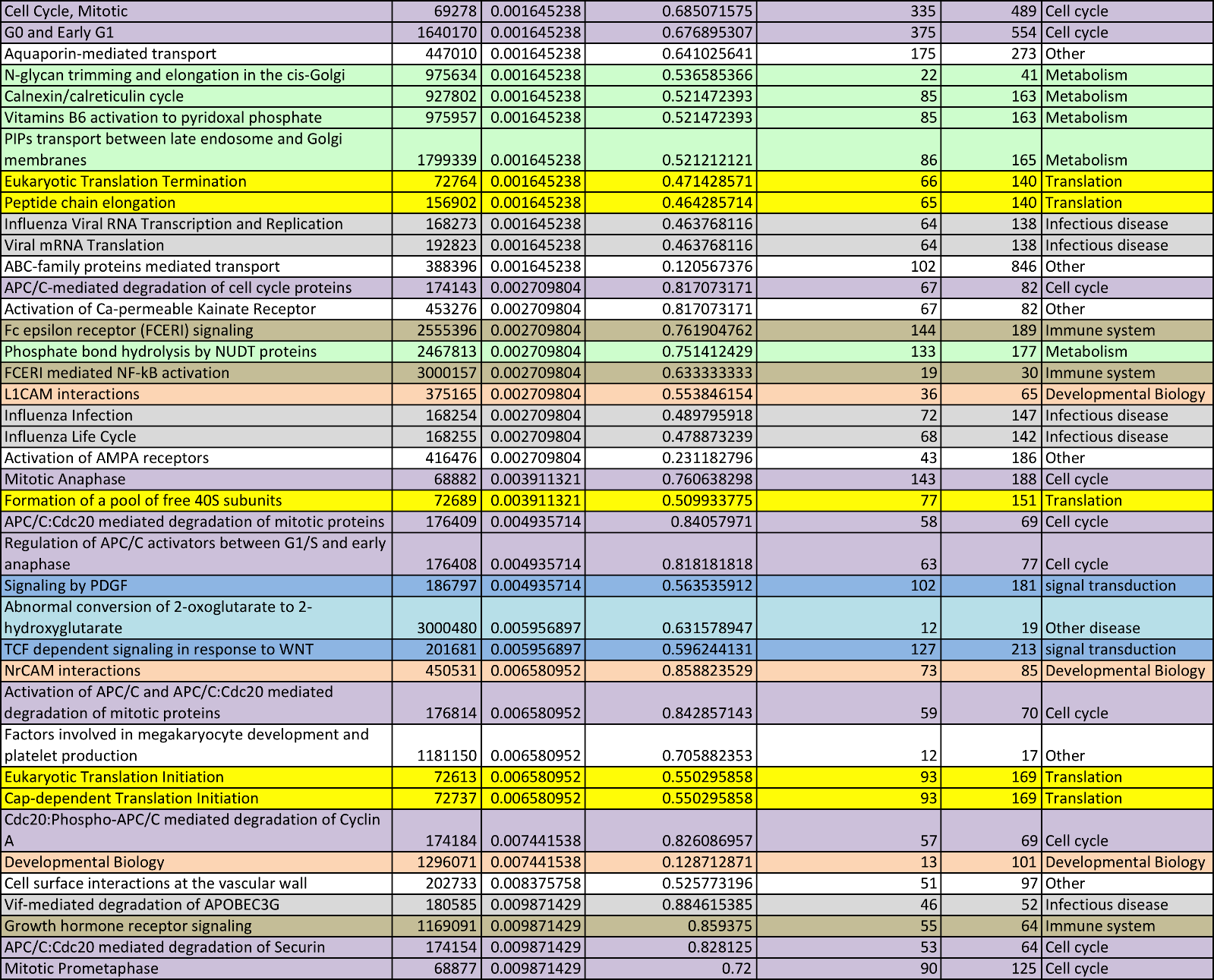


**Table S3. Statistically significant pathway terms from one-group *pathVar* analysis using the SD for the Yan single human embryonic stem cells profiled at passage 0 (adjusted P-value < 0.01). A.** KEGG pathways and **B.** REACTOME terms.

**A.** KEGG


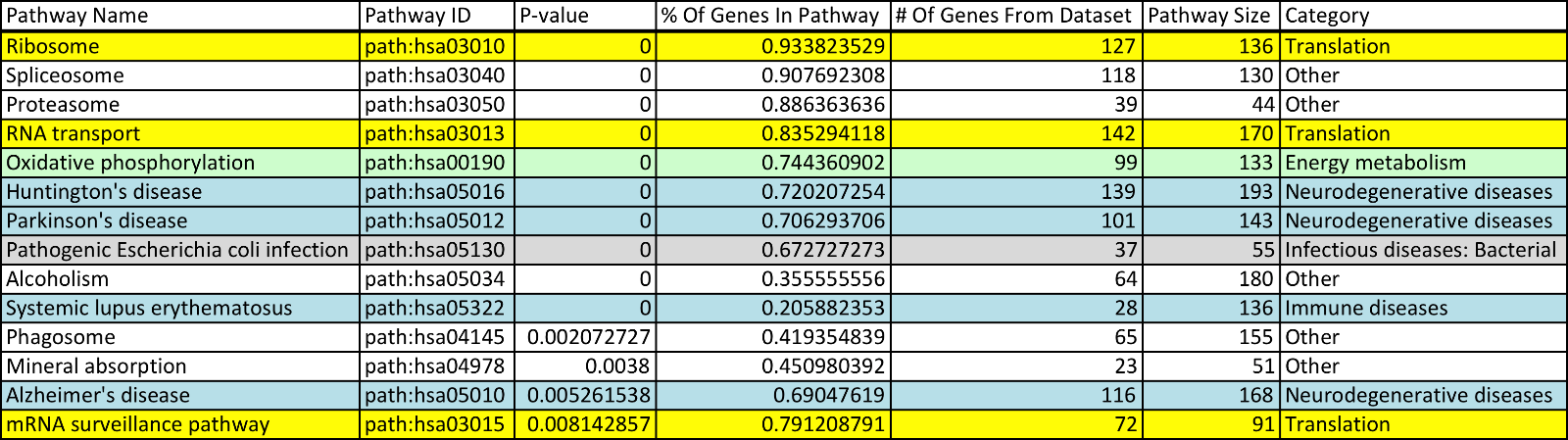


**B.** REACTOME.


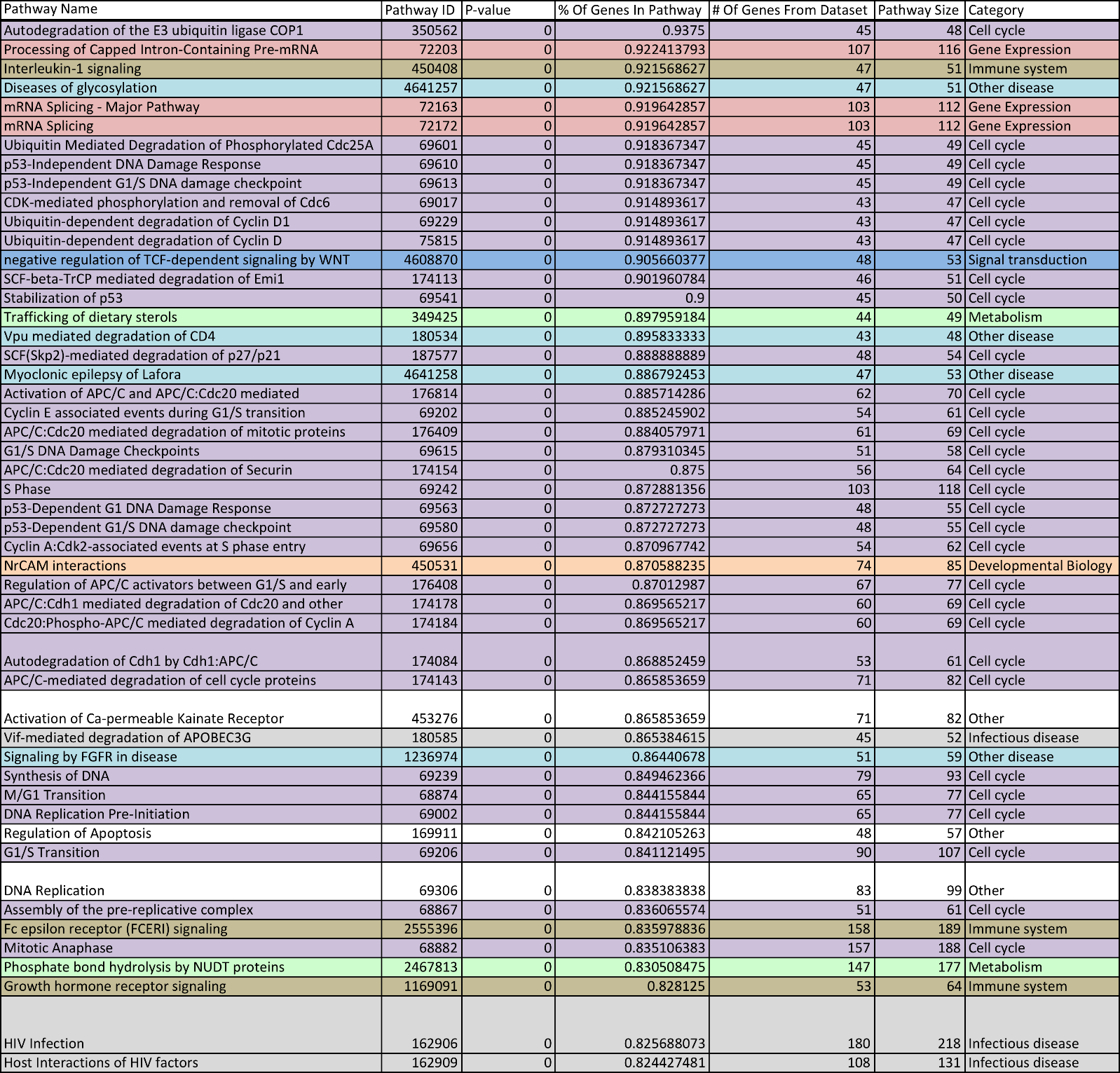


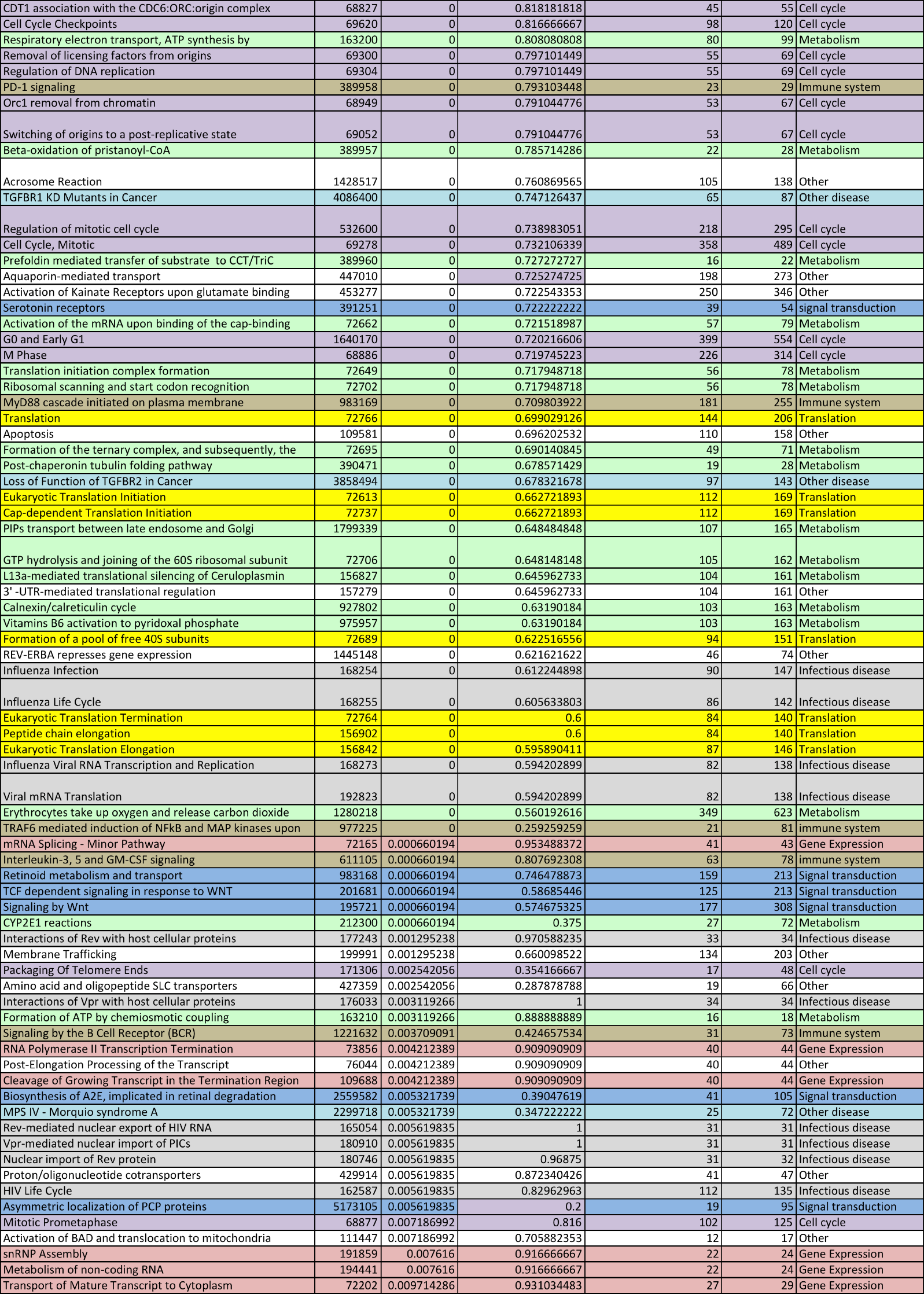


**Table S4. Statistically significant pathway terms from one-group *pathVar* analysis using the SD for the Yan single human embryonic stem cells profiled at passage 10 (adjusted P-value < 0.01). A.** KEGG pathways and **B.** REACTOME terms.

**A.** KEGG.


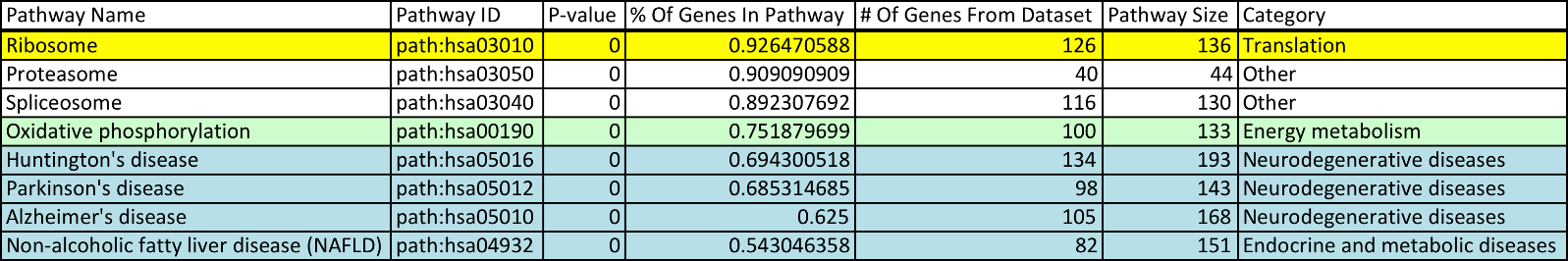


**B.** REACTOME.


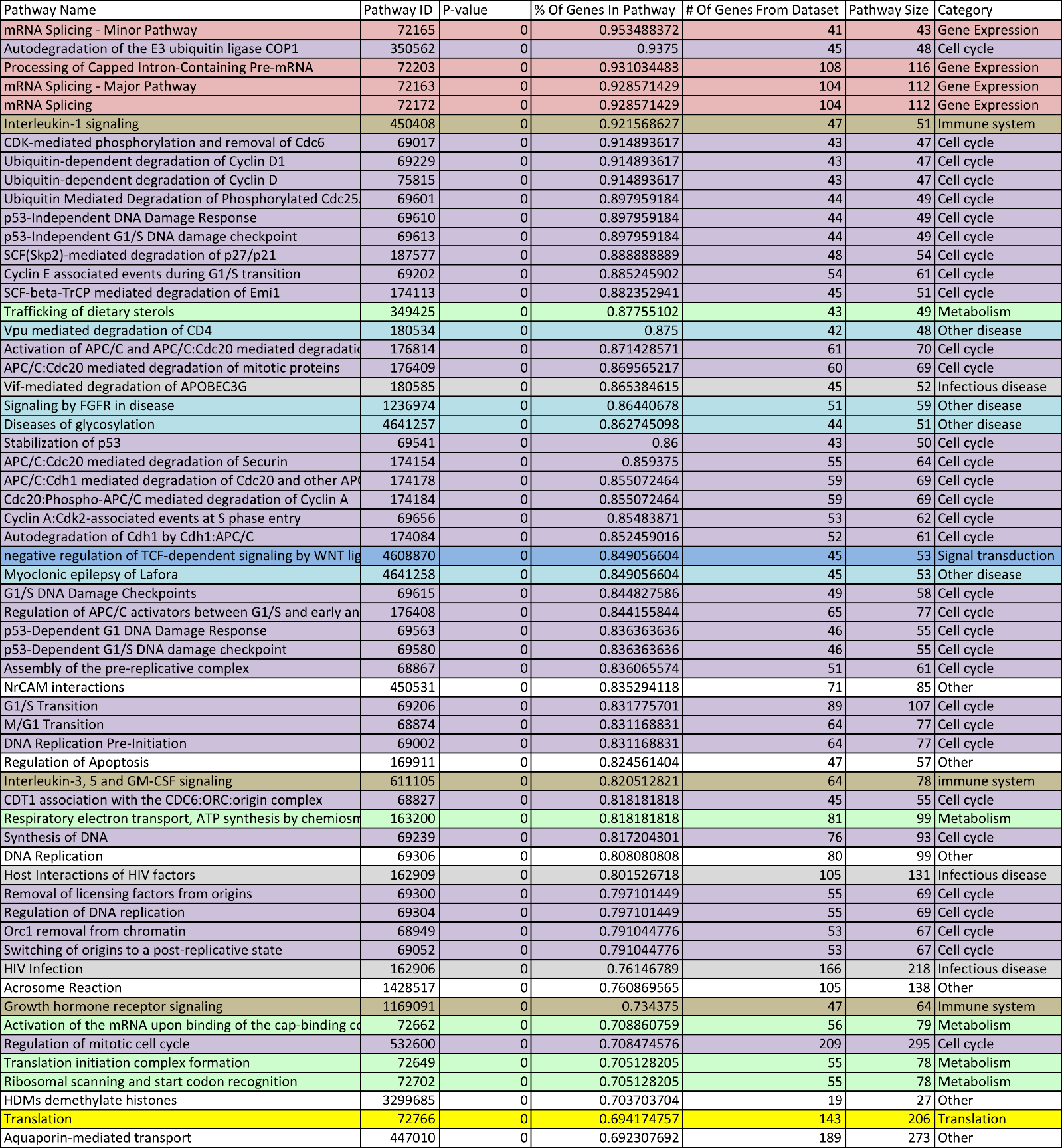


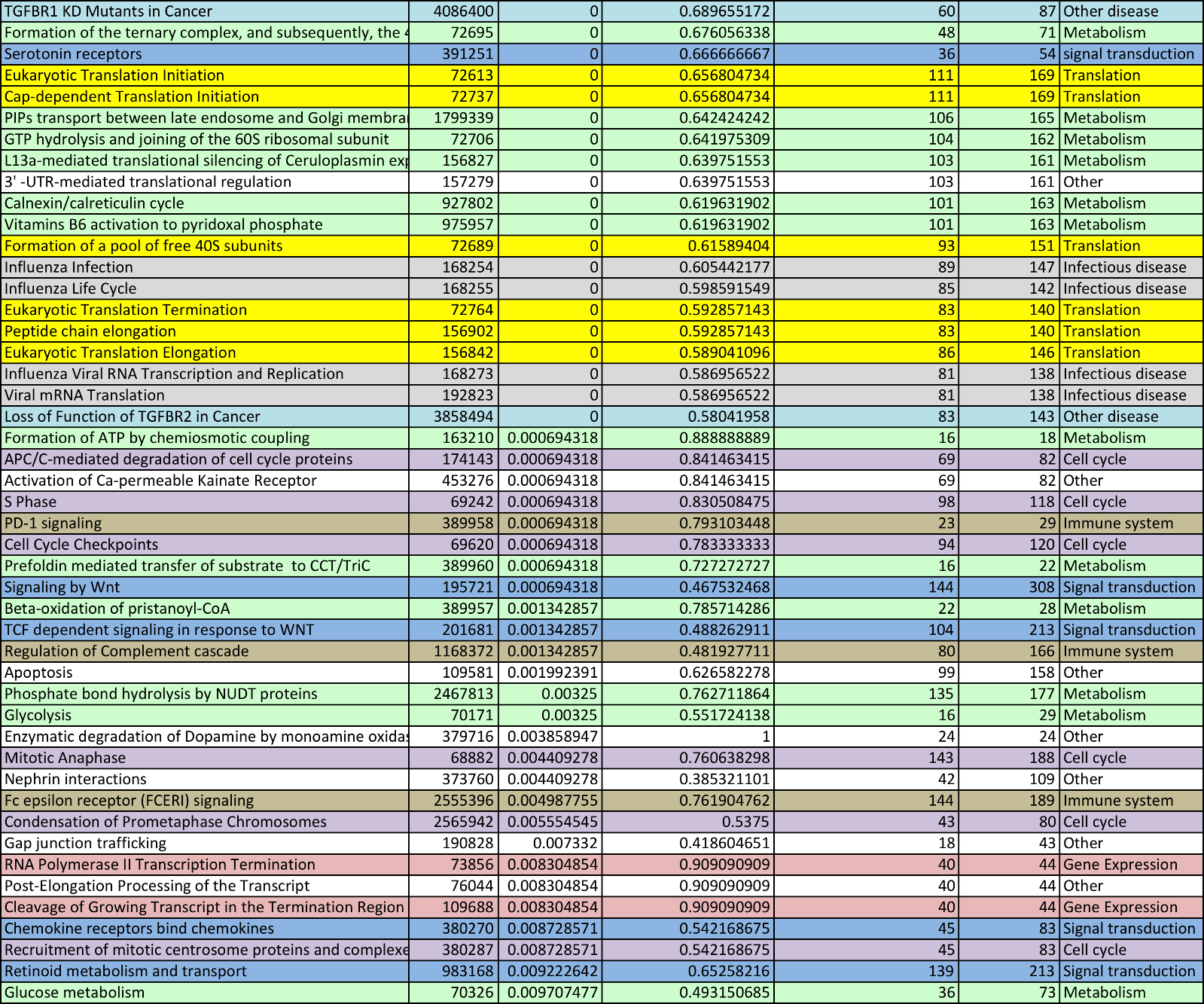


**Table S5. Number of significant pathways obtained for the ESC versus iPSC two-group case (adjusted P-value < 0.01).** Number of significant pathways obtained for **A.** KEGG and **B.** REACTOME in the two-group case when using *pathVar* based on either the variability statistic, or average gene expression. We also report the number of significant pathway terms that were identified in both the mean-based and variability-based analysis.

| Pathway Database | Comparison | Samples | Genes | Significant for variability (SD) | Significant for mean | Intersection mean/variability |
| --- | --- | --- | --- | --- | --- | --- |
| KEGG | ESC vs iPSC | 20 ESCs, 12 iPSCs | 7564 | 6 | 1 | 0 |
| REACTOME | ESC vs iPSC | 20 ESCs, 12 iPSCs | 7564 | 30 | 3 | 1 |

**Table S6. Statistically significant pathways with a change in gene expression variability using *pathVar* based on the SD between human ESCs and iPSCs from the Bock data set (adjusted P-value < 0.01).**

**A.** KEGG.


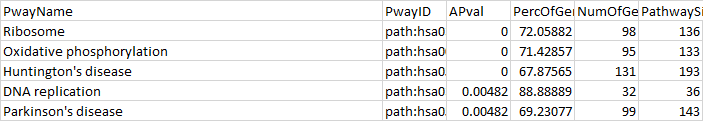


**B.** REACTOME.


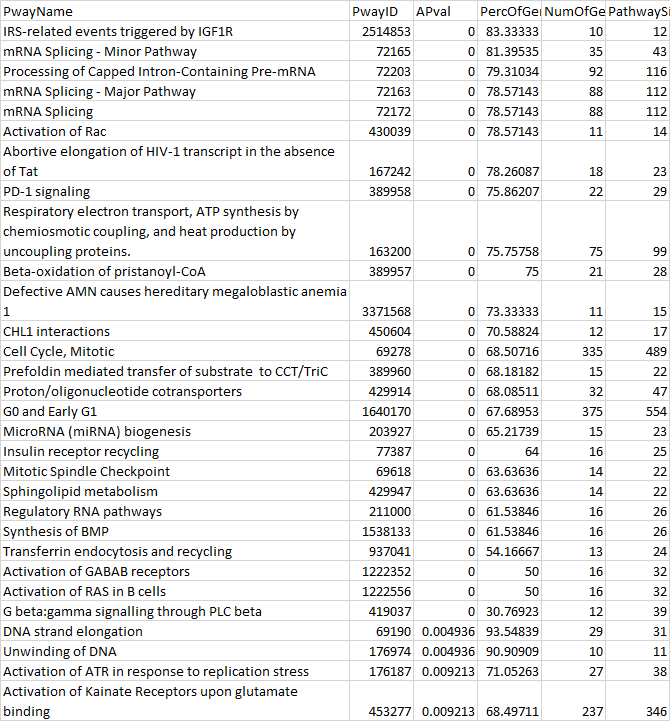


**Table S7.** **Statistically significant pathways with a change in average gene expression using *pathVar* based on average expression between human ESCs versus iPSCs from the Bock data set (adjusted P-value < 0.01).**

**A.** KEGG.


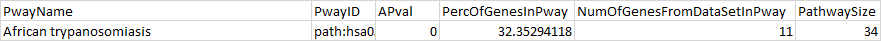


**B.** REACTOME.


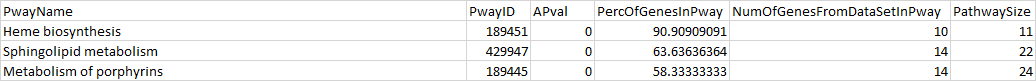


**Table S8. Number of significant pathways obtained for all gene expression datasets for the one-group case (adjusted P-value < 0.01).** Number of significant pathways obtained for **A.** KEGG and **B.** REACTOME in the one-group case when using *pathVar* based on either the variability statistic, or average gene expression. We also report the number of significant pathway terms that were identified in both the mean-based and variability-based analysis.

**A.** KEGG

| Data Sets | Type | Genes | Samples | Variability | Significant for Variability | Significant for Mean | Intersection Mean ∩ Variability |
| --- | --- | --- | --- | --- | --- | --- | --- |
| TCGA AML | RNA-seq | 14681 | 173 | MAD | 80 | 130 | 48 |
| TCGA GBM | RNA-seq | 16216 | 169 | SD | 93 | 141 | 60 |
| TCGA OVC | RNA-seq | 16187 | 309 | MAD | 96 | 117 | 58 |
| Yan hESC | Single cell RNA-seq | 6667 | 34 | SD | 11 | 14 | 8 |
| Bock hESC | Microarray | 7632 | 20 | SD | 25 | 24 | 9 |
| Bock iPSC | Microarray | 7646 | 12 | SD | 19 | 21 | 9 |
| 1000 Genomes | RNA-seq | 10206 | 660 | SD | 19 | 68 | 8 |
| Host Malaria | Microarray | 24891 | 98 | CV | 34 | 145 | 30 |
| Parasite Malaria | Microarray | 5199 | 56 | CV | 16 | 60 | 14 |
| Mouse Hippocampus | Microarray | 18138 | 100 | CV | 4 | 99 | 4 |
| Mouse Striatum | Microarray | 18138 | 98 | CV | 2 | 92 | 2 |
| Down syndrome iPSC | Microarray | 21040 | 12 | SD | 9 | 102 | 4 |
| Wild-type iPSC | Microarray | 21040 | 15 | SD | 11 | 115 | 6 |

**B.** REACTOME

| Datasets | Type | Genes | Samples | Variability | Significant for variability | Significant for mean | Intersection mean/variability |
| --- | --- | --- | --- | --- | --- | --- | --- |
| AML | RNA-seq | 14681 | 173 | MAD | 347 | 396 | 248 |
| GBM | RNA-seq | 16216 | 169 | SD | 424 | 439 | 291 |
| OVC | RNA-seq | 16187 | 309 | MAD | 351 | 447 | 287 |
| Yan hESC | Single cell RNA-seq | 6667 | 34 | SD | 95 | 131 | 94 |
| Bock hESC | Microarray | 7632 | 20 | SD | 65 | 138 | 43 |
| Bock iPSC | Microarray | 7646 | 12 | SD | 66 | 149 | 42 |
| 1000 Genomes | RNA-seq | 10206 | 660 | SD | 18 | 217 | 7 |
| Host Malaria | Microarray | 24891 | 98 | CV | 22 | 387 | 22 |
| Down syndrome iPSC | Microarray | 21040 | 12 | SD | 31 | 437 | 20 |
| Wild-type iPSC | Microarray | 21040 | 15 | SD | 38 | 478 | 27 |

**Table S9. The overlap in significant pathways obtained for all gene expression datasets for the two-group case (adjusted P-value < 0.01) between different statistics.** The tables report the number of significant pathways obtained for **A.** KEGG and **B.** REACTOME in the two-group case when using *pathVar* based on either the variability statistic, average gene expression, and contrasted against two implementations of GSEA, GSEAlm (Oron et al.) and based on the Kolmogorov-Smirnov test using limma P-values (see Results).We also report the number of significant pathway terms that were commonly identified between the variability-based approach using *pathVar*, and the other methods using average expression, and the two GSEA-based implementations.

**A.** KEGG

| *Comparison* | AML vs 1000 Genomes | GBM vs 1000 Genomes | OVC vs 1000 Genomes | Bock hESC vs iPSC | Down Syndrome vs Wild-type iPSCs | Mouse Hippocampus vs Striatum |
| --- | --- | --- | --- | --- | --- | --- |
| **Number of Genes** | 8944 | 8970 | 8986 | 7554 | 7000 | 18138 |
| **Variability Statistic** | MAD | MAD | MAD | MAD | SD | CV |
| **Significant Variability Pathways** | 182 | 126 | 169 | 8 | 1 | 117 |
| **Significant Mean Pathways** | 248 | 243 | 246 | 1 | 1 | 0 |
| ***Intersection of Mean ∩ Variability*** | 182 | 121 | 167 | 0 | 0 | 0 |
| **Significant for GSEAlm** | 296 | 296 | 296 | 0 | 37 | 197 |
| **Intersection of GSEAlm ∩ Variability** | 181 | 125 | 168 | 0 | 0 | 72 |
| **Significant for GSEA (limma P-value)** | 26 | 25 | 26 | 2 | 2 | 14 |
| **Intersection of GSEA (limma P-value) ∩ Variability** | 25 | 22 | 24 | 1 | 0 | 8 |

**B.** REACTOME

| *Comparison* | AML vs 1000 Genomes | GBM vs 1000 Genomes | OVC vs 1000 Genomes | Bock hESC vs iPSC | Down Syndrome vs Wild-type iPSCs |
| --- | --- | --- | --- | --- | --- |
| **Number of Genes** | 8944 | 8970 | 8986 | 7554 | 7000 |
| **Variability Statistic** | MAD | MAD | MAD | MAD | SD |
| **Significant Variability Pathways** | 447 | 296 | 344 | 39 | 57 |
| **Significant Mean Pathways** | 714 | 711 | 713 | 3 | 12 |
| ***Intersection of Mean ∩ Variability*** | 446 | 292 | 342 | 1 | 1 |
| **Significant for GSEAlm** | 1186 | 1188 | 1189 | 0 | 187 |
| **Intersection of GSEAlm ∩ Variability** | 263 | 179 | 204 | 0 | 5 |
| **Significant for GSEA (limma P-value)** | 169 | 32 | 24 | 3 | 4 |
| **Intersection of GSEA (limma P-value) ∩ Variability** | 24 | 8 | 8 | 0 | 0 |

**Table S10. Top ten statistically significant KEGG pathways (adjusted P-value < 0.01) in the two-group cancer versus normal comparisons.** We report the top ten pathways or all the significant pathways if there were less than ten for **A.** AML vs 1000 Genomes, **B.** GBM vs 1000 Genomes, **C.** OVC vs 1000 Genomes. Blue cells denote terms that were observed for both the variability-based and average expression-based *pathVar* analyses. Yellow cells denote terms that were unique to either the variability-based or average expression-based *pathVar* analysis.

**A.** AML vs 1000 Genomes.

MAD


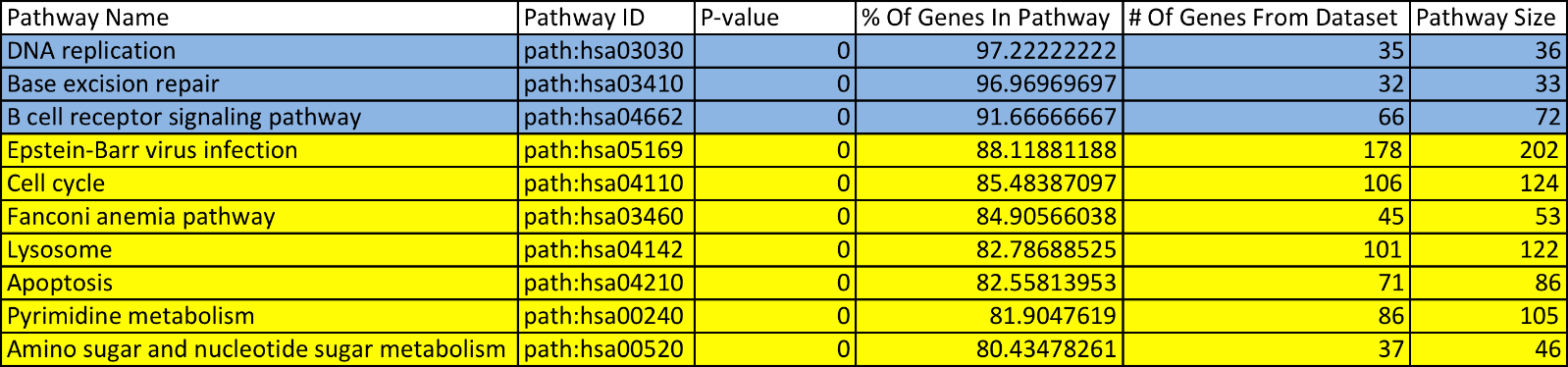


Mean


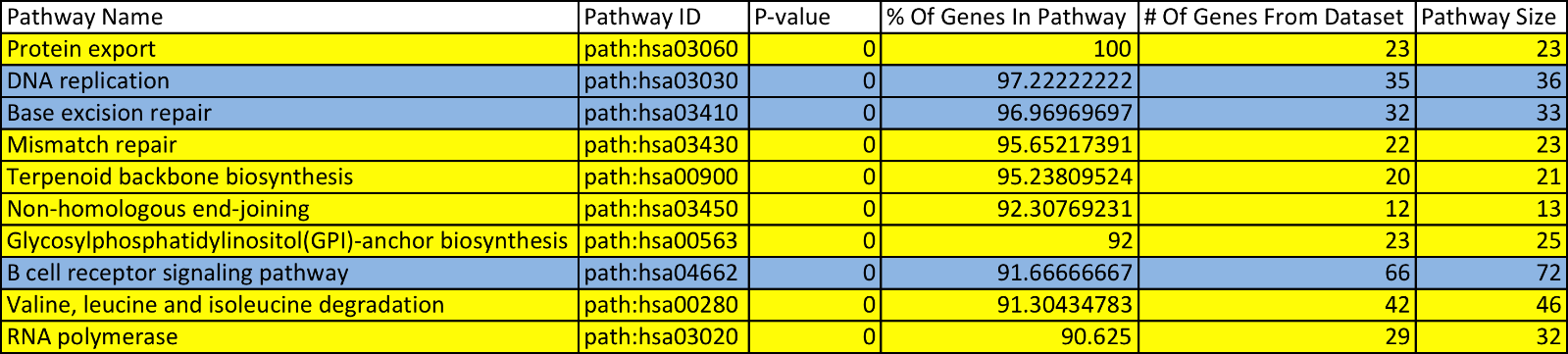


**B.** GBM vs 1000 Genomes

MAD


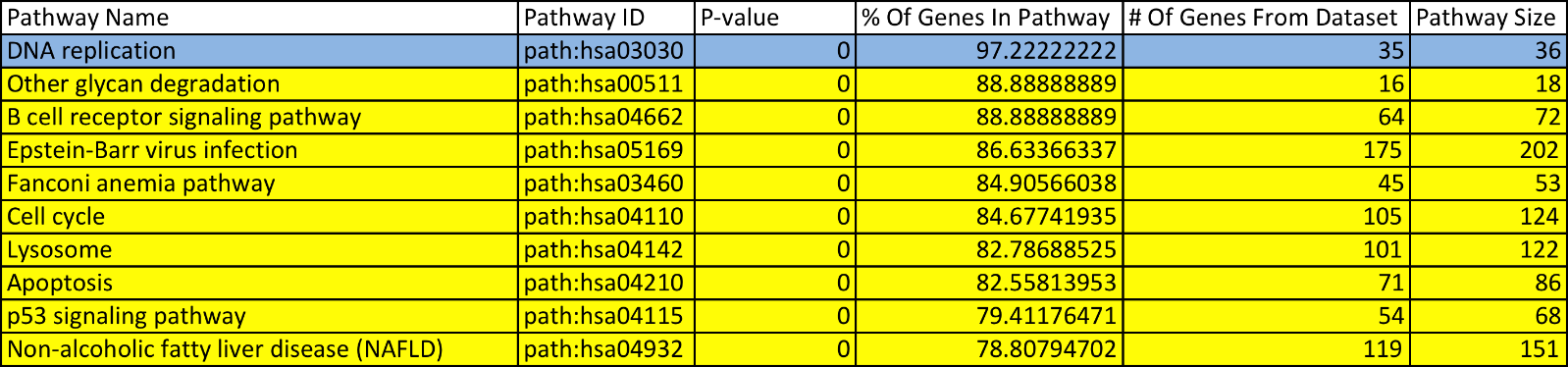


Mean


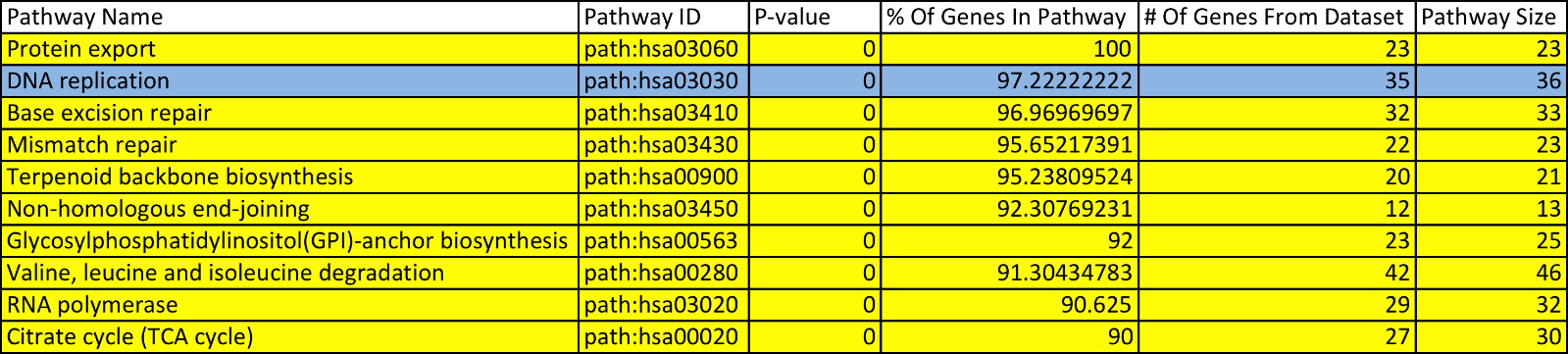


**C.** OVC vs 1000 Genomes

MAD


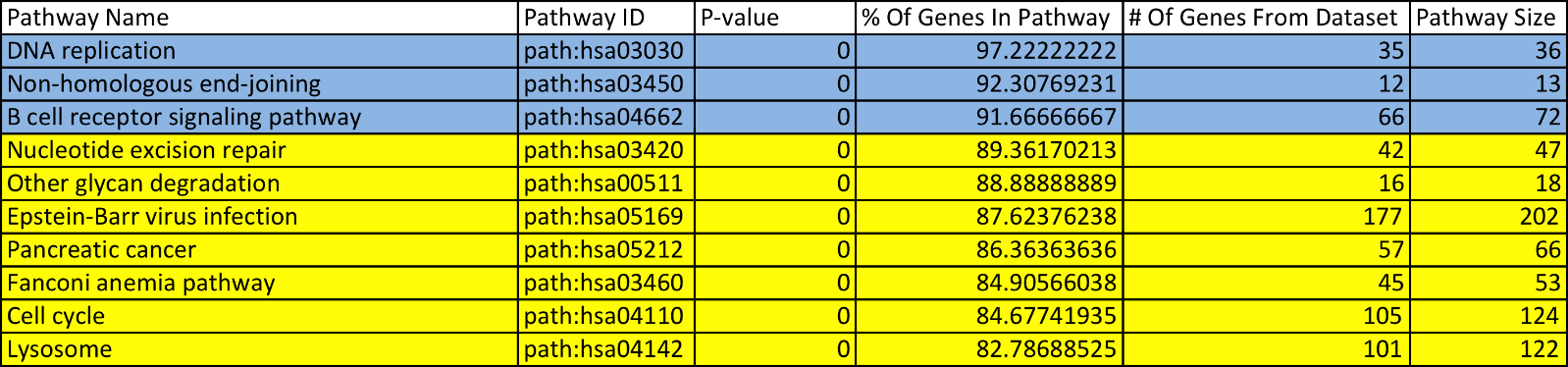


Mean


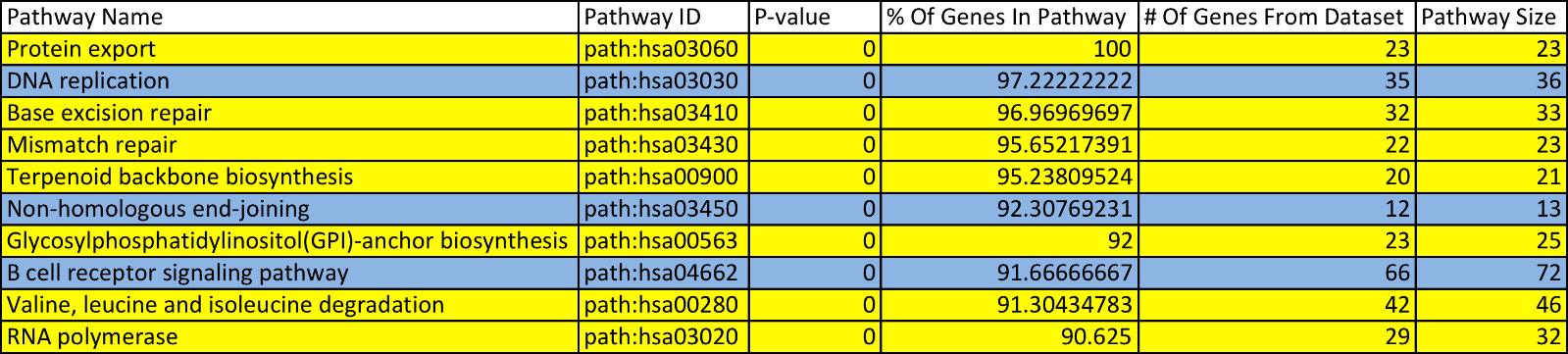


**Table S11. Top ten statistically significant REACTOME terms (adjusted P-value < 0.01) in the two-group cancer versus normal comparisons.** We report the top ten pathway terms or all the significant pathways if there were less than ten for **A.** AML vs 1000 Genomes, **B.** GBM vs 1000 Genomes, **C.** OVC vs 1000 Genomes. Blue cells denote terms that were observed for both the variability-based and average expression-based *pathVar* analyses. Yellow cells denote terms that were unique to either the variability-based or average expression-based *pathVar* analysis.

**A.** AML vs 1000 Genomes.

MAD


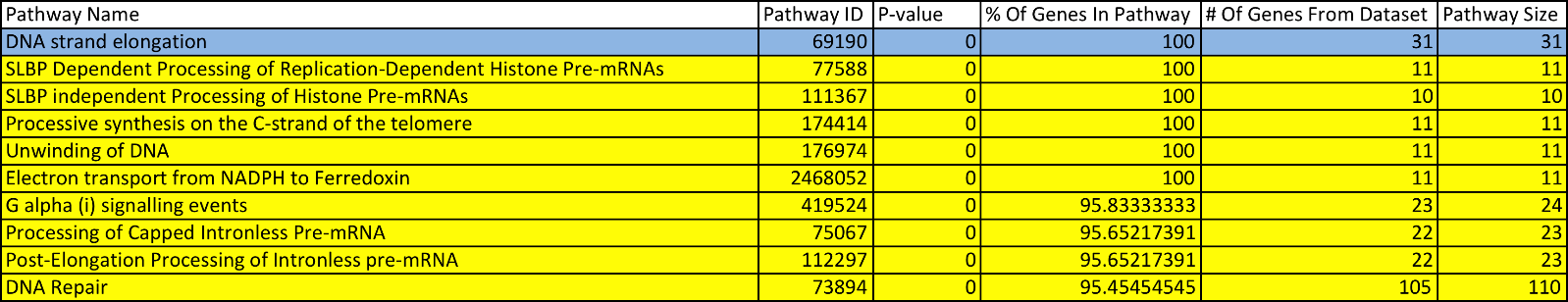


Mean


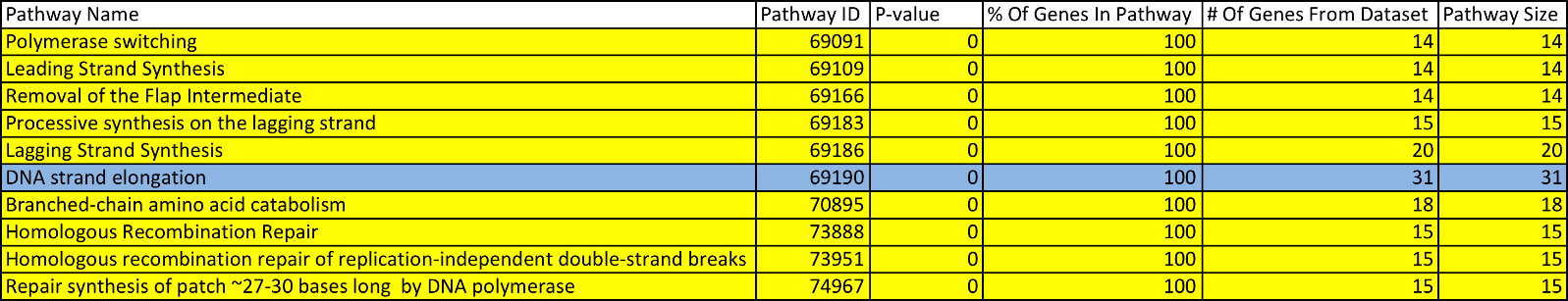


**B.** GBM vs 1000 Genomes.

MAD


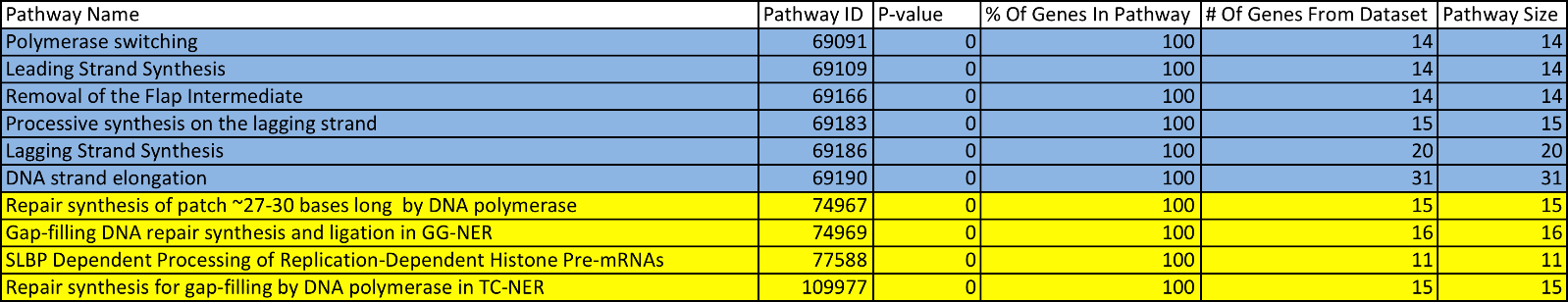


Mean


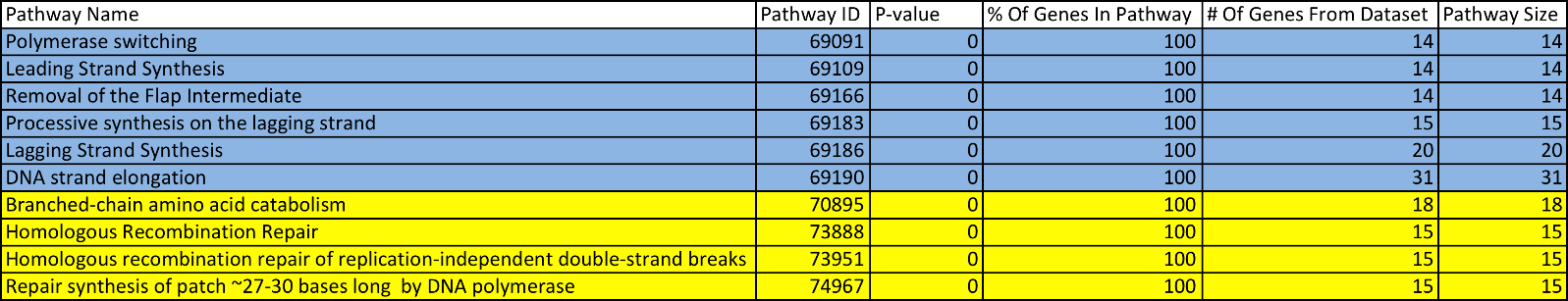


**C.** OVC vs 1000 Genomes.

MAD


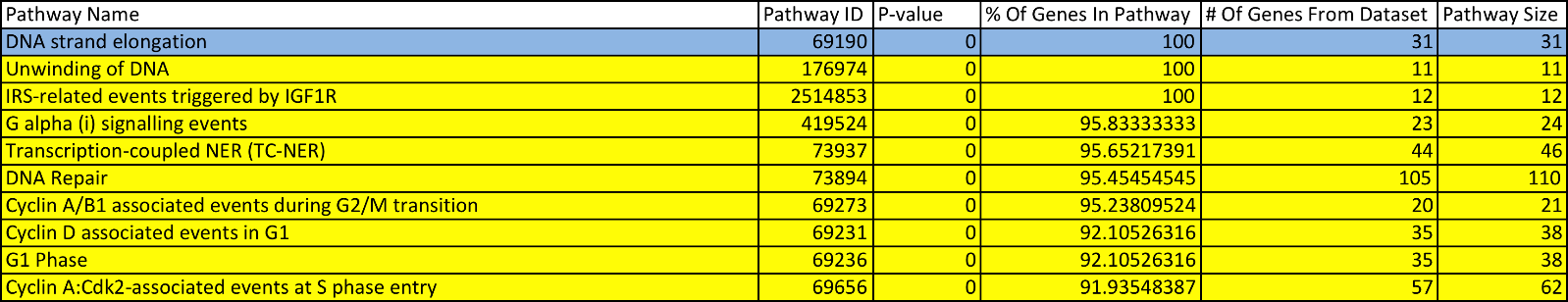


Mean


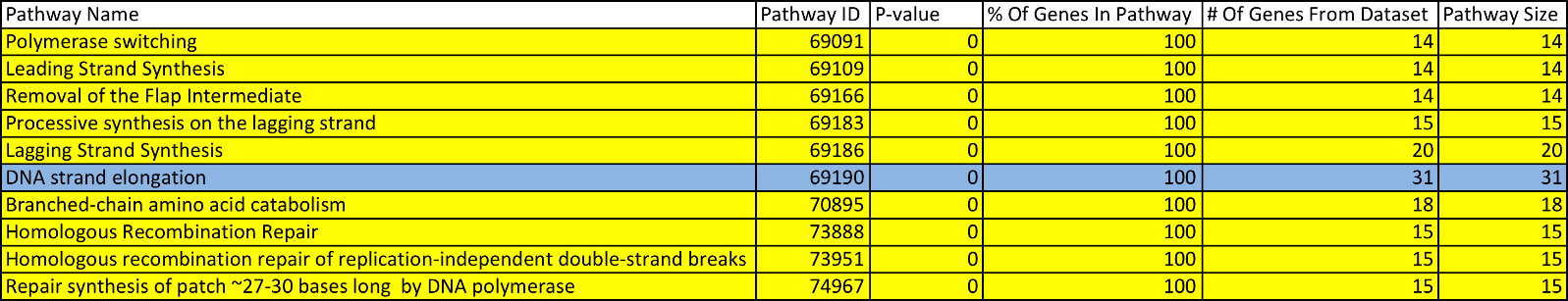


**Table S12. Assessing the overlap in significant pathways detected by different approaches in the presence of varying degrees of dependency between the average expression and expression variability in the data.**

| *Comparison* | AML vs 1000 Genomes | GBM vs 1000 Genomes | OVC vs 1000 Genomes | Bock hESC vs iPSC | Down Syndrome vs Wild-type iPSCs | Mouse Hippocampus vs Striatum |
| --- | --- | --- | --- | --- | --- | --- |
| **Correlation Coefficient between Variability and Average** | 0.064 | 0.039 | 0.22 | -0.182 | -0.405 | 0.239 |
| **KEGG**  **Intersection of GSEAlm ∩ Variability** | 181 | 125 | 168 | 0 | 0 | 72 |
| **KEGG**  **Intersection of GSEA (limma P-value) ∩ Variability** | 25 | 22 | 24 | 1 | 0 | 8 |
| **REACTOME**  **Intersection of GSEAlm ∩ Variability** | 263 | 179 | 204 | 0 | 5 | - |
| **REACTOME**  **Intersection of GSEA (limma P-value) ∩ Variability** | 24 | 8 | 8 | 0 | 0 | - |

***Supplementary Texts***

**Text S1: Simulations and Power Calculations to Test Parameters of the *pathVar* Method**

**Text S2: Pre-processing Steps Applied to the Stem Cell Gene Expression Data Sets**

**Text S3: Application of *pathVar*using the Variability Statistic versus Average Expression on Ten Different Gene Expression Datasets**

**Text S4. Comparison of Trends in Variability between bulk versus single cell data.**
