# Supplementary material for: pathVar: a new method for pathway-based interpretation of gene expression variability
Source: PeerJ. 2017 May 23;5:e3334. doi: 10.7717/peerj.3334 (PMC5444375; doi:10.7717/peerj.3334)
Supplement: Text S1 [file peerj-05-3334-s002.docx]

**Text S1: Simulations and Power Calculations to Test Parameters of the *pathVar* Method**

**Investigating the performance of the three variability statistics.** To investigate which of the three variability statistics had the best performance in *pathVar*, data sets under 18 different scenarios were simulated (Figure S1.1A). The simulations were designed around the one-group case where gene expression data were generated under one of three discrete variance categories reflecting low, medium and high levels of gene expression variability. The different scenarios mimic a variety of situations where the variances of the three levels are quite similar or different, the means are also similar or different or a combination of the two (Figure S1.1B). The proportion of total genes in each of the different levels of variable and mean expression were varied to reflect equal or skewed distributions (Figure S1.2). Two tests were applied to evaluate the performance of the SD, CV and MAD in the *pathVar* method under this extensive simulation framework.

**Testing a variability estimator’s ability to classify genes into their correct levels of expression variability.** For the first test, we evaluated how accurately genes in the simulation were able to be assigned to their true levels of low, medium and high gene expression variability when the data was summarized using the different estimators. As part of the first step of the *pathVar* method, genes were assigned to discrete variability categories using the mixture model algorithm *mclust* [13]. We specified the correct model of three clusters or mixtures with unequal variance in *mclust* and then calculated the average misclassification rate observed across 100 simulated data sets for each of the 18 different simulation scenarios (Table 1.1, Figure S1.3). For all three estimators, the misclassification rates increased overall as the difference in variance between the discrete groups got smaller (parameter set *v_3_*, Figure S1.1B). The SD however had consistently the lowest average misclassification rate for all 18 different scenarios.

**Testing a variability estimator’s ability to identify the correct model of expression variability.** For the second test, we investigated how accurately *mclust* could identify the correct model when no information was specified for each of the different variability statistics. When a model is not specified in advance in *mclust*, the algorithm fits a range of models to the data and uses the Bayesian Information Criteria (BIC) as a means of evaluating which model has the most appropriate fit for the data. For each of the 18 different scenarios, we counted how many times out of the 100 data sets that were simulated, the model of three clusters with unequal variance was correctly identified (Table 1.2, Figure S1.4). Although the performance was more varied across the different scenarios, and not as unanimous as the first test, overall we saw that when using the MAD, the correct model was specified the most often compared to the other two estimators (Figure S1.4).

**Analysis of power and effect size.** The power and effect size of the Chi-squared test were computed using the R package pwr (version 1.1.3). Simulations demonstrate that for a fixed effect size, the power of the Chi-squared test increases when the size of a pathway also increases (Figure S1.5). Similarly, when we consider a pathway of a fixed size, the power of the test is higher for a larger effect size. To investigate how power is affected in *pathVar* by different kinds of gene expression variability distributions, consider an example where the reference distribution is defined as *p_0_* = (0.25, 0.5, 0.25) where the low variability genes represent 25% of all genes, the medium variability genes represent 50% of all genes, and the high variability genes represent 25% of all genes. What would be the power of the Chi-squared test to detect the difference between a pathway and a reference distribution where the pathway has a count distribution defined by *p_1_* = (0.35, 0.4, 0.25) or *p_2_* = (0.3, 0.4, 0.3) (see Figure S1.6)? Pathway *p_1_* represents an example where the distribution is weighted towards more low variability genes, while pathway *p_2_* has a symmetric distribution where the ratio of low to high variability genes is equal.

**Figure S1.1. Outline of the 18 different scenarios that gave rise to the simulated data sets. A.** The parameter sets that defined each of the different data scenarios. **B.** The qualitative features underlying each of the parameters to provide motivation for its inclusion in the simulation study.

**A.**


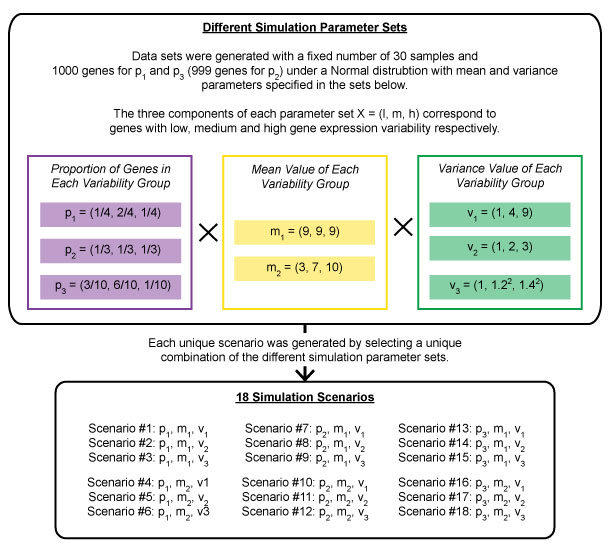


**B.**


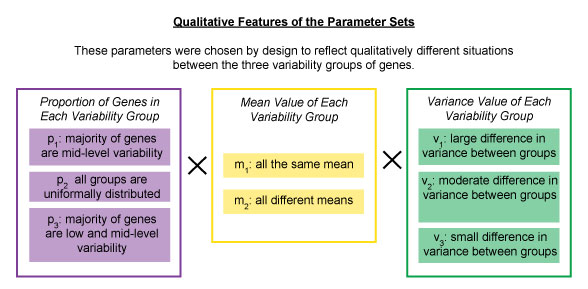


**Figure S1.2. Variability count distributions of the simulated data sets generated under the 18 different scenarios.** The distributions are represented as **A.** density plots showing how the three groups of low, medium and high variability genes differ from each other with respect to population statistics, and **B.** histogram bar plots displaying the different weights between the three variability groups.

**A.**


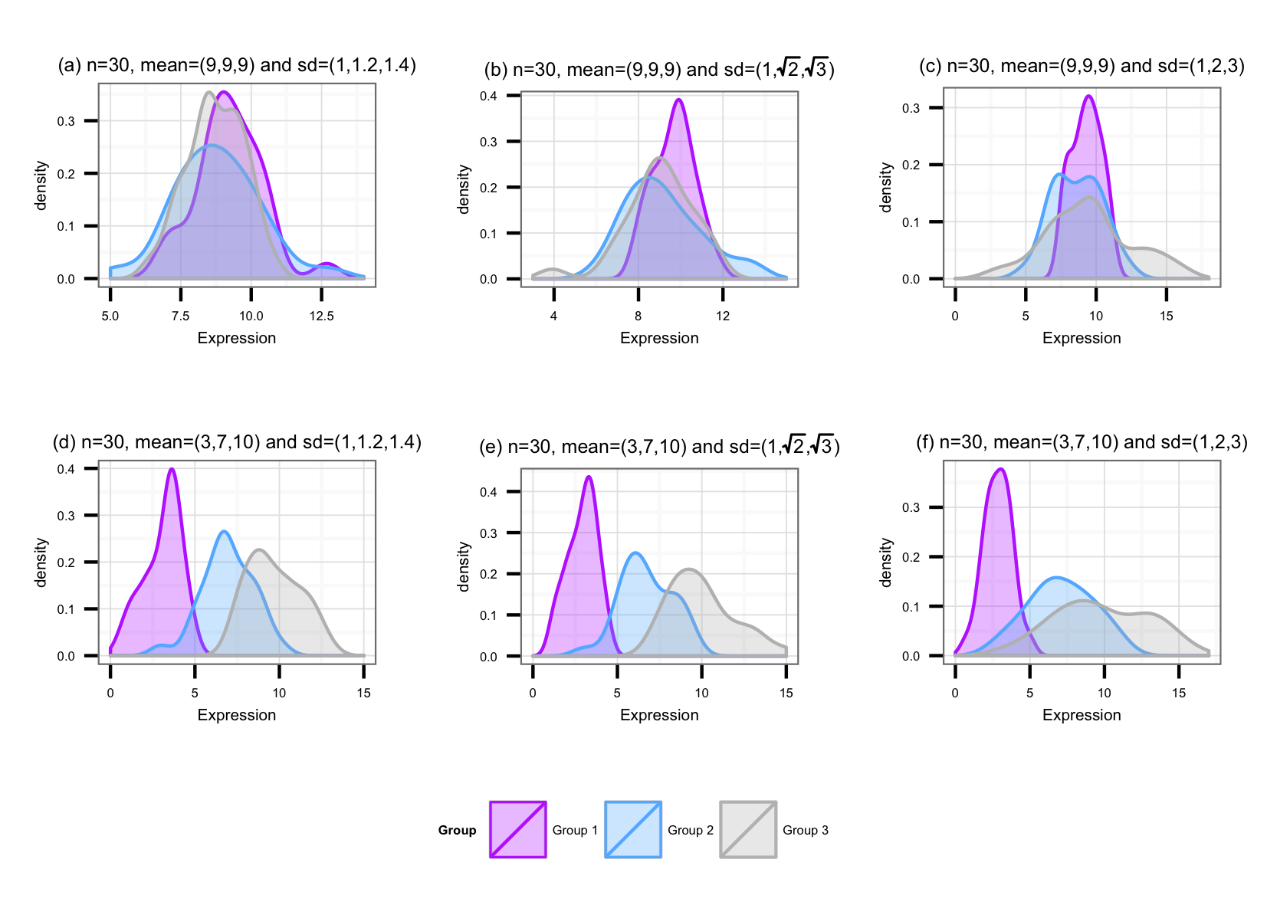


**B.**


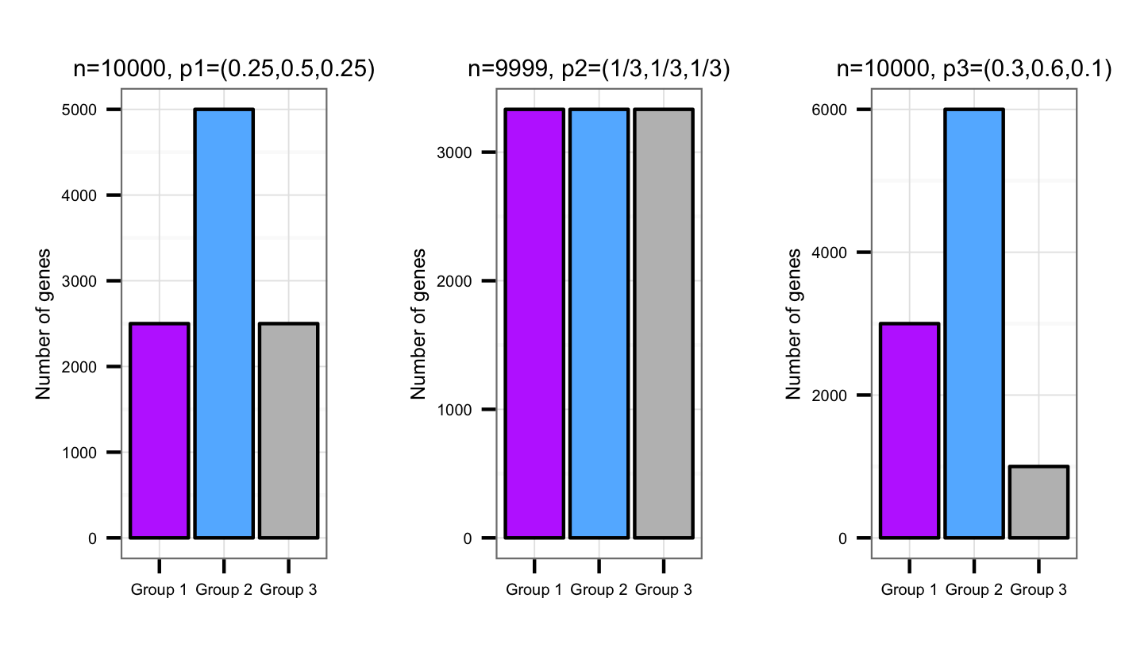


**Figure S1.3. Evaluating the performance of the SD, MAD and CV under the 18 different scenarios based on assigned of genes to their correct variability levels.** Boxplots representing the number of misclassified genes in each of three variability categories (low, medium or high). The definition of the parameters P1, P2, P3, M1, M2, V1, V2, V3 can be found in Figure S1.

**Figure S1.4. Evaluating the performance of the SD, MAD and CV under the 18 different scenarios based on selection of the correct variance model using mclust.** Boxplots of the BIC resulting from *mclust* were evaluated for each of the eight candidate models (2V indicates a model with two mixtures or clusters, with unequal variance; 2E indicates a model with two mixtures or clusters, with equal variance). The definition of the parameters P1, P2, P3, M1, M2, V1, V2, V3 can be found in Figure S1.

**Figure S1.5. The power of the Chi-squared test increases for larger effect sizes.** Given a fixed number of genes, the power of the Chi-squared test is larger for larger effect sizes. Similarly, for a larger number of genes, the power of the test increases.


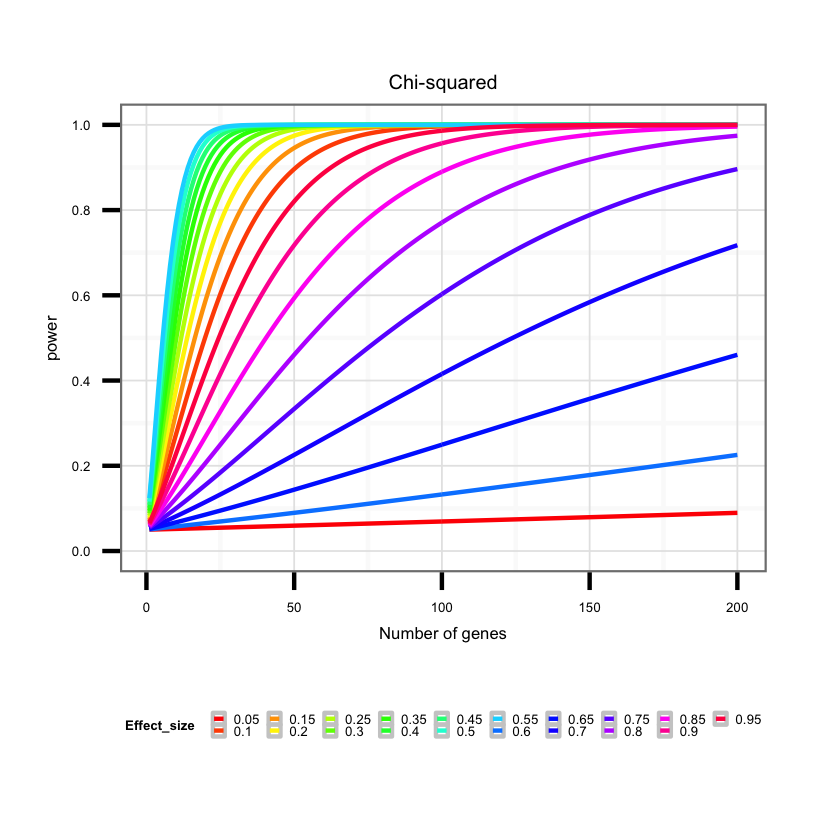


**Figure S1.6. Examples of how different variability count distributions affect the power of the Chi-squared test.** **A.** The reference distribution, shown in gray, is defined as *p_0_* = (0.25, 0.5, 0.25) representing the percentage of low, medium and high variability genes, respectively. The count distributions for the two example pathways are defined as **B.** *p_1_* = (0.35, 0.4, 0.25) and **C.** *p_2_* = (0.3, 0.4, 0.3) in blue where *p_1_* is skewed towards lower levels of expression variability, and *p_2_* is symmetrically distributed. **D.** When comparing the power of the Chi-squared test, which assesses the difference between the pathway and reference distributions, we see that the power is higher for *p_1_* than *p_2_* given a fixed number of genes in a pathway. The red line corresponds to a power = 0.8.


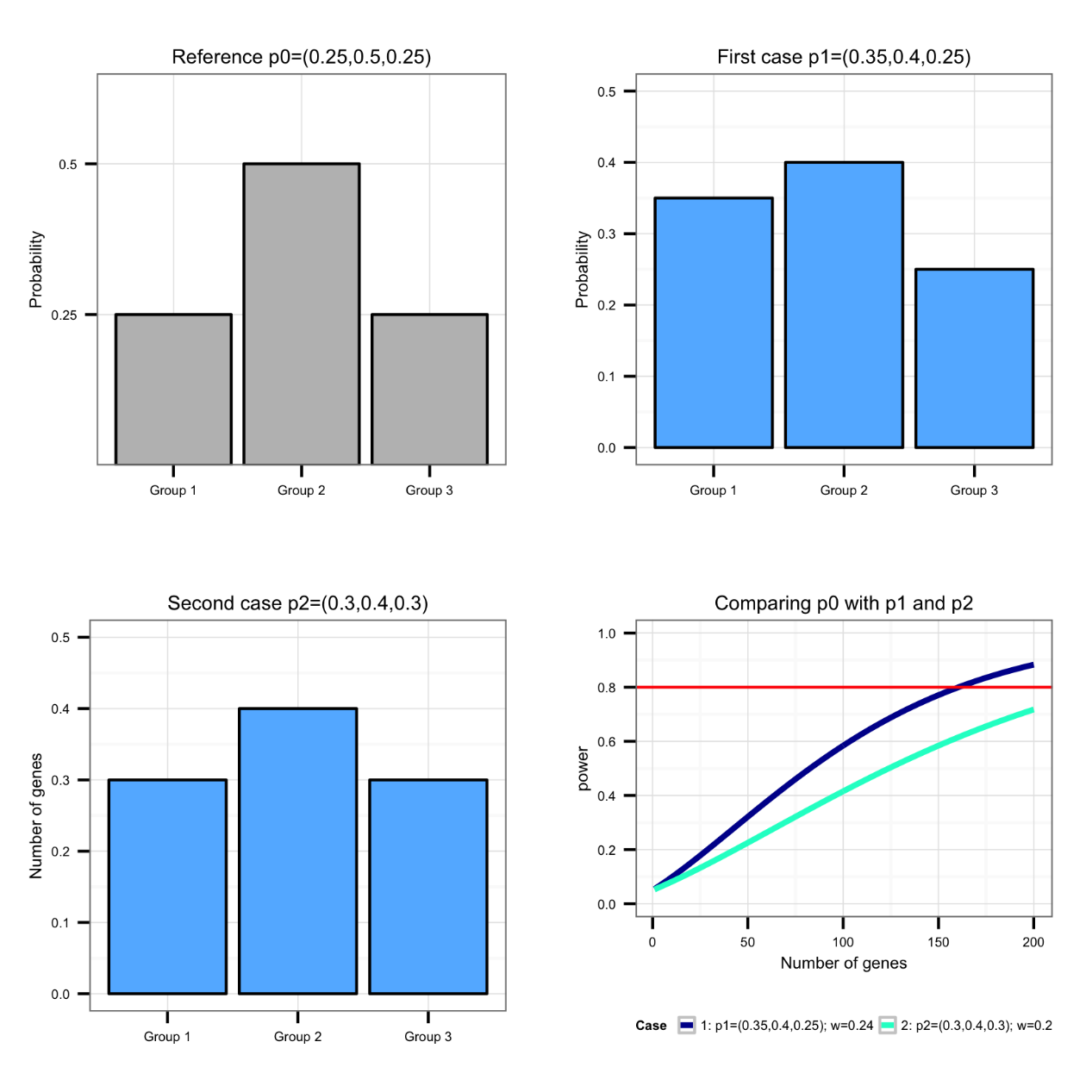


**Figure S1.7. Investigating the effect of power and number of samples in the data set on the Chi-squared test.** A simulation was designed to evaluate the power calculations stemming from a range of effect sizes, for a Chi-squared test at 0.05 level of significance with a fixed number of genes in each simulation set that was selected to reflect small to large pathways. **A.** 50 genes, **B.** 100 genes, **C.** 500 genes, **D.** 1000 genes.


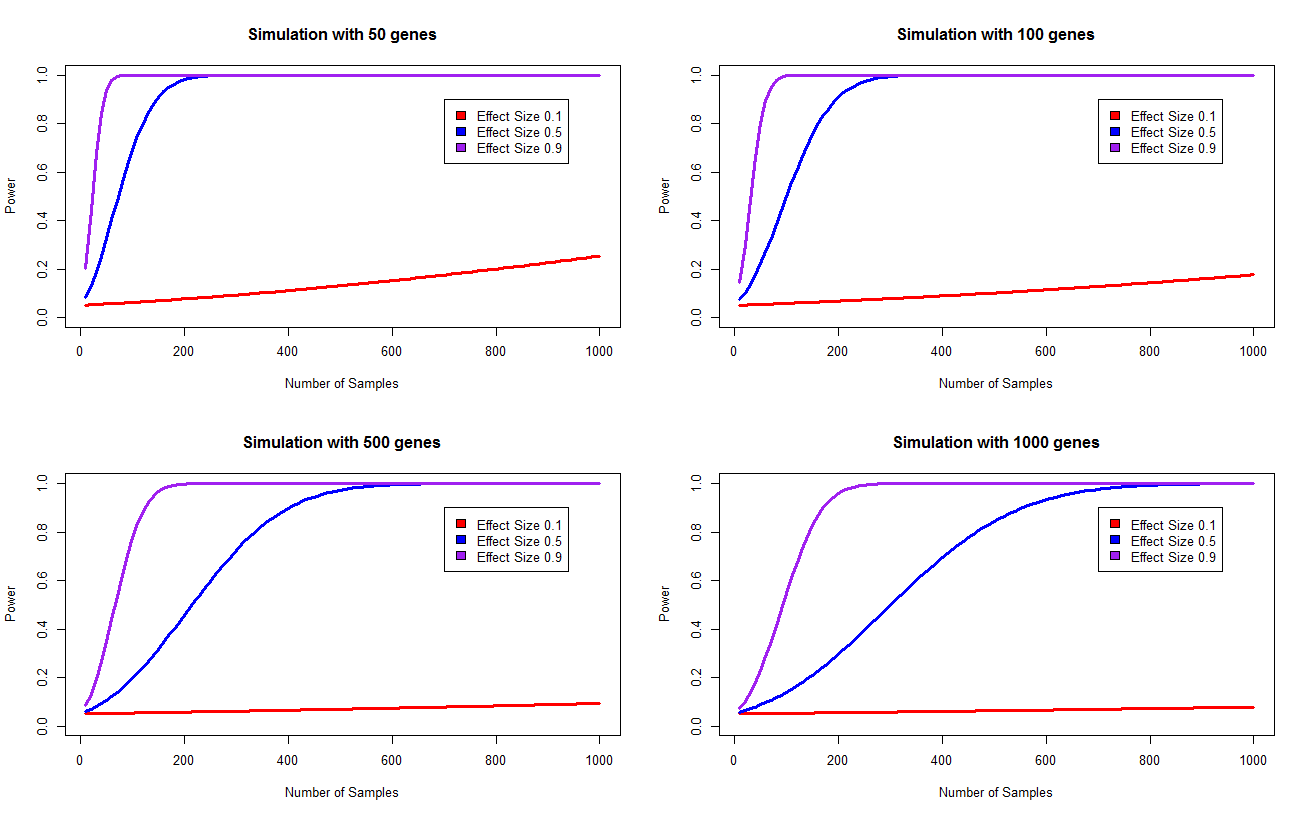


**Table S1.1. Results from data simulations to test the performance of different variability estimators - when the correct model is specified, how many genes are misclassified?** Gene expression data were simulated for 18 different scenarios, described in Figure S1. Under each of the experimental designs specified for a scenario, 100 replicate data sets were generated. The correct model (three clusters, unequal variance) was specified in *mclust*, and the average number of genes that were misclassified are reported. The total number of genes for P1 and P3 was 10,000 and for P2 was 9999. The standard deviation of the number of misclassified genes is reported in parentheses.

|  |  |  | Misclassification mean (SD) when the model with three clusters and unequal variance is specified | | | | | |
| --- | --- | --- | --- | --- | --- | --- | --- | --- |
|  |  |  | SD (Number) | SD (%) | MAD (Number) | MAD (%) | CV (Number) | CV (%) |
| P1 | M1 | V1 | 480.08 (26.03) | 4.80 | 1661.37 (45.39) | 16.61 | 669.99 (53.28) | 6.70 |
|  |  | V2 | 2435.12 (97.78) | 24.35 | 3915.16 (69.59) | 39.15 | 2491.5 (68.03) | 24.92 |
|  |  | V3 | 4043.08 (91.85) | 40.43 | 4933.54 (95.13) | 49.34 | 4074.45 (65.53) | 40.75 |
|  | M2 | V1 | 482.57 (26.98) | 4.83 | 1669.11 (51.77) | 16.69 | 7217.96 (61.34) | 72.18 |
|  |  | V2 | 2430.4 (93.94) | 24.30 | 3898.75 (86.76) | 38.99 | 6872.16 (57.85) | 68.72 |
|  |  | V3 | 4056.89 (91.13) | 40.57 | 4920.9 (92.11) | 49.21 | 6495.24 (52.02) | 64.95 |
| P2 | M1 | V1 | 458.21 (19.94) | 4.58 | 1544.39 (41.21) | 15.45 | 565.2 (27.98) | 5.62 |
|  |  | V2 | 2130.72 (97.78) | 21.31 | 3538.47 (47.67) | 35.39 | 2168.8 (38.25) | 21.69 |
|  |  | V3 | 3517.81 (50.09) | 35.18 | 4666.44 (60.16) | 46.67 | 3549.54 (41.77) | 35.50 |
|  | M2 | V1 | 460.63 (20.91) | 4.61 | 1539.96 (35.82) | 15.40 | 7566.78 (53.67) | 75.68 |
|  |  | V2 | 2137.91 (40.82) | 21.38 | 3533.24 (51.75) | 35.34 | 7920.18 (40.81) | 79.21 |
|  |  | V3 | 3528.75 (60.35) | 35.29 | 4667.46 (56.72) | 46.68 | 7589.6 (37.00) | 75.90 |
| P3 | M1 | V1 | 321.26 (33.37) | 3.21 | 1198.97 (47.11) | 11.99 | 461.1 (37.59) | 4.61 |
|  |  | V2 | 2103.21 (204.66) | 21.03 | 2186.91 (208.43) | 21.87 | 2583.47 (155.07) | 25.84 |
|  |  | V3 | 4206.99 (216.41) | 42.07 | 4805.59 (384.54) | 48.06 | 4475.1 (162.53) | 44.75 |
|  | M2 | V1 | 316.36 (28.77) | 3.16 | 1190.76 (45.65) | 11.91 | 7145.5 (103.65) | 71.46 |
|  |  | V2 | 2139.24 (214.80) | 21.39 | 2154.21 (186.29) | 21.54 | 6853.62(67.85) | 68.54 |
|  |  | V3 | 4192.77 (204.69) | 41.93 | 4776.64 (363.06) | 47.77 | 6551.67 (63.42) | 65.52 |

**Table S1.2. Results from data simulations to test the performance of different variability estimators – when the model is not specified, how many times can the correct model be identified?** Using the same simulated data sets, we counted how many times (out of a total of 100) *mclust* correctly identified the model under different variability estimators. 3V indicates a model with three clusters and unequal variance that was identified by *mclust*, whereas 3E indicates the same model but with equal variance. The correct model that the data was simulated under was 3V.

|  |  |  | Number of clusters and equal (E) or unequal (V) variances over 100 simulations | | |
| --- | --- | --- | --- | --- | --- |
|  |  |  | SD | MAD | CV |
| P1 | M1 | V1 | 3V: 100 | 3V: 100 | 3V: 100 |
|  |  | V2 | 3V: 100 | 3V: 95; 4V: 5 | 3V: 100 |
|  |  | V3 | 2V: 32; 3V: 3, 3E: 65 | 2V: 33; 3V: 62, 4V: 1, 3E: 1, 4E: 3 | 2V: 79; 3V: 16, 2E: 1, 3E: 4 |
|  | M2 | V1 | 3V: 100 | 3V: 100 | 2V: 75; 3V: 2, 2E: 21, 4E: 2 |
|  |  | V2 | 2V: 1, 3V: 99 | 3V: 94, 4V: 4, 3E: 1, 5E:1 | 2V: 24, 3V: 76 |
|  |  | V3 | 2V: 28, 3V: 2, 2E: 1, 3E: 69 | 2V: 28, 3V: 70, 3E: 1, 4E: 1 | 3V: 100 |
| P2 | M1 | V1 | 3V: 100 | 3V: 100 | 3V: 100 |
|  |  | V2 | 2V: 87, 3V: 13 | 3V: 97, 4V: 3 | 2V: 96, 3V: 4 |
|  |  | V3 | 2V: 80, 3V: 3, 3E: 17 | 2V: 22, 3V: 74, 3E: 3, 4E: 1 | 2V: 46, 3V: 34, 3E: 20 |
|  | M2 | V1 | 3V: 100 | 3V: 100 | 2V: 84, 2E: 15, 4E: 1 |
|  |  | V2 | 2V: 89, 3V: 11 | 3V: 99, 4V: 1 | 2V: 4, 3V: 96 |
|  |  | V3 | 2V: 82, 3V: 4, 3E: 14 | 2V: 18, 3V: 78, 3E: 2, 4E: 2 | 3V: 100 |
| P3 | M1 | V1 | 3V: 100 | 3V: 100 | 3V: 100 |
|  |  | V2 | 3V: 99, 3E: 1 | 3V: 100 | 2V: 1, 3V: 99 |
|  |  | V3 | 2V: 27, 3V: 17, 2E: 7, 3E: 42, 4E: 7 | 2V: 38, 3V: 56, 3E: 2, 4E: 4 | 2V: 56, 3V: 37, 4E: 7 |
|  | M2 | V1 | 3V: 100 | 3V: 100 | 2V: 75, 3V: 3, 2E: 22 |
|  |  | V2 | 3V: 97, 4E: 3 | 3V: 99, 4E: 1 | 2V: 98, 3V: 2 |
|  |  | V3 | 2V: 26, 3V: 15, 2E: 4, 3E: 46, 4E: 9 | 2V: 37, 3V: 61, 3E: 2 | 2V: 100 |
