# Supplementary material for: pathVar: a new method for pathway-based interpretation of gene expression variability
Source: PeerJ. 2017 May 23;5:e3334. doi: 10.7717/peerj.3334 (PMC5444375; doi:10.7717/peerj.3334)
Supplement: Text S2 [file peerj-05-3334-s003.docx]

**Text S2: Pre-processing Steps Applied to the Stem Cell Gene Expression Data Sets**

All data sets featured in this paper are publicly accessible from GEO. Where possible, the pre-processing steps were standardized across different data sets. Here, we include the specific details of the pre-processing steps in order to facilitate reproducibility, as well as some results to illustrate the motivation for applying the pre-processing steps to the data.

**Gene expression datasets.** The ESCs and iPSCs from the Bock data set can be accessed via GEO under the accession number GSE25970, and the Yan data set is stored under GSE36552.

**Initial pre-processing steps.** All data was log_2_-transformed and normalized. For all data sets, a quality filter was applied to remove genes that had RPKM < 1 in more than 25% of the samples profiled. Upon inspection of the expression densities for the RNA-seq data (Figure S2.1) it was clear that the RPKM = 1 threshold corresponded to a second mode of transcripts that were likely not expressed or lowly expressed in the data. After filtering, 7622 genes were retained for the ESC from the Bock dataset, 8459 and 6248 genes from p0 and p10 in the Yan dataset that will be used for the one sample case. For the two samples cases, there were 7554 genes belonging to both ESC and iPSC datasets (see Table S2.1).

**Analysis of human ESCs and iPSCs.** In both cases, *pathVar* was run using the SD as the variability statistic and the exact multinomial test to identify pathways with aberrant expression variability distributions. The variability statistic was chosen after evaluation of the correlation between the three variability statistics versus the average expression for the four different data sets. It was apparent that the MAD and SD had the lowest correlation (Figure S2.2). While the MAD had the lower correlation than the SD for three of the four data sets, the difference was minimal (Table S2.2), therefore we chose to use SD in our analysis since downstream interpretation of this statistic was easier than the MAD.

**Figure S2.1. Justifying a low-level gene expression filter to pre-process the data sets.** Inspection of the global distribution of gene expression obtained for the human embryonic stem cell (ESC) line data shows two populations of genes in the **A.** Yan data set for single cells profiled at passage 0 and passage 10 with 20,286 transcripts, and **B.** the ESCs and iPSCs in the Bock data set with 12,889 genes. Based on the bimodal distribution, genes are either expressed or not expressed. The latter group was removed as it is likely that these genes have such low expression signal that they will not contribute to the analysis and may represent noise. We used a threshold of log_2_(expression) = 1 as the cut-off.

**A.** Yan data sets.

**B.** Bock data sets.

**Figure S2.2. Choosing a variability estimator based on its correlation between variability and average gene expression.** Scatter plots demonstrate the degree of correlation between the variability estimator and the average expression where the solid line represents the loess curve that shows the non-linear smoothed, trend of the data, and R represents the Pearson correlation coefficient. **A.** The Bock ESC data set with 20 cell lines based on 7,622 genes. **B.** The Bock iPSC data set with 11 cell lines based on 7,636 genes, **C.** The Yan data set with 8 single ESCs at passage 0 based on 8,459 genes, **D.** The Yan data set with 26 single ESCs at passage 10 based on 6,248 genes.

**A.** Bock data set for the 20 ESCs.

**B.** Bock data set for the 12 iPSCs.

**C.** Yan data set for the 8 single ESCs at passage 0.

**D.** Yan data set for the 26 single ESCs at passage 10.

**Table S2.1. Keeping track of the genes filtered out during pre-processing of the data sets.** Using a cut-off of log_2_(normalized expression) = 1, genes were filtered out of the analysis if they did not meet this threshold. We record the number of genes that were removed for **A.** the Bock data sets, and the **B.** Yan data sets.

**A.** Bock data sets.

|  | hESCs (n=20) | iPSCs (n=12) |
| --- | --- | --- |
| Genes with > 1 in at least 75% of samples | 7622 | 7636 |
| Genes with > 1 in at least 75% of samples in hESCs and iPSCs | 7554 | |

**B.** Yan data sets.

|  | p0 (n=8) | p10 (n=26) |
| --- | --- | --- |
| Genes with > 1 in at least 75% of samples | 8459 | 6248 |

**Table S2.2. Different variability estimators and their correlation coefficients with average gene expression.** After lowly-expressed genes were filtered out, we calculated the Pearson correlation coefficient of the SD, MAD and CV with average expression for **A.** the Bock data set, and **B.** the Yan data set.

**A.** Bock data set.

|  | SD | MAD | CV |
| --- | --- | --- | --- |
| ESC | -0.234 | -0.190 | -0.627 |
| iPSC | -0.238 | -0.174 | -0.625 |

**B.** Yan data set.

|  | SD | MAD | CV |
| --- | --- | --- | --- |
| p0 | -0.46 | -0.393 | -0.744 |
| p10 | -0.43 | -0.525 | -0.842 |
