# Supplementary material for: pathVar: a new method for pathway-based interpretation of gene expression variability
Source: PeerJ. 2017 May 23;5:e3334. doi: 10.7717/peerj.3334 (PMC5444375; doi:10.7717/peerj.3334)
Supplement: Text S3 [file peerj-05-3334-s004.docx]

**Text S3: Application of *pathVar* using the Variability Statistic versus Average Expression on Ten Different Gene Expression Datasets**

To highlight the generalizability of *pathVar*, we selected ten other datasets that cover a variety of biological and experimental variables. Collectively, these ten datasets were generated from multiple technology platforms that featured samples from human, mouse and parasite which represent a range of different disease phenotypes.

Of the diseases selected, three cancer RNA-seq datasets were used from the Cancer Genome Atlas (TCGA) ; these were the ovarian serous cystadenocarcinoma (OVC) [20], acute myeloid leukemia (AML) [21], and glioblastoma multiforme (GBM) [22] patient cohorts. An infectious disease was included where transcriptomes from patients infected with cerebral malaria were profiled using microarrays [23], as well as the *Plasmodium falciparum* parasites that the patients were infected with [24]. We included a genetic disorder, where patient-derived iPSCs were collected from Down syndrome (DS) donors and profiled using microarrays, with a set of matched controls from healthy subjects [25]. A microarray dataset from a normal human population via the Geuvadis study [26] (1000 Genomes Project [27]) was used, as well as two mouse datasets that profiled tissues from two different regions of the brain, the hippocampus and the striatum using microarrays [28]. *pathVar* identified statistically significant pathways from KEGG and REACTOME pathway terms for the one-group analysis (Table S3.1), and two-group comparisons (Table S3.2) of all ten datasets.

**Gene expression datasets.** For the Cancer Genome Atlas data, Level 3 Illumina HiSeq RNA-seq version 2 data were downloaded from <http://cancergenome.nih.gov/> for acute myeloid leukemia (AML), ovarian serious cystadenocarcinoma (OVC), and glioblastoma multiforme (GBM). The AML dataset had 173 patient samples, the GBM data had 169 patient samples and the OVC data had 309 patient samples; all three datasets had 20531 genes. The Geuvadis/1000 Genomes dataset was downloaded from http://www.ebi.ac.uk/Tools/geuvadis-das/. The data from the patients infected with cerebral malaria are available under the accession number GSE9152, and the parasite transcriptomes are under GSE72058. The mouse data is available under the accession number GSE26500. The Down syndrome dataset is stored under GSE42956.

**Initial pre-processing steps.** All data was log_2_-transformed and normalized. GBM and OVC were batch-corrected using the function ComBat from the R package **sva** (version 3.14.0). For all two-group comparisons, the intersection of genes between both datasets were used.

**Benchmarking results from *pathVar* using the average expression versus the variability statistic identifies significant pathways that provide unique functional insights into regulation of phenotypes.** We used the ten different datasets as a means to investigate the uniqueness of results obtained with analyses based on average expression versus gene expression variability. The average-based version of *pathVar* was run concurrently for each dataset comparison, and we calculated the number of pathways that had significant changes in both mean and variability. For all cases, we noticed that the amount of overlap in significant pathways differed depending on the datasets that were used, suggesting that average and variability-based statistics reflect different ways in which cells may use their transcription programs depending on the biological context (Table S3.1 & S3.2). For the KEGG pathways, *pathVar* results for the DS versus WT iPSC comparison, and the mouse hippocampus versus striatum comparison both had zero overlap between average-based and variability-based significant pathways. In fact, the latter comparison also yielded no significant pathways with a difference in average, whereas 116 KEGG pathways were found to have a difference in gene expression variability. These two comparisons are extreme examples where the analyses of gene expression variability identify changes in the transcriptional program, whereas average-based analyses do not yield significant results.

Overall, it was apparent that the transcriptional features responsible for distinguishing one phenotype from another are exerted through changes in average expression or variability in expression for key pathways. To further investigate the relevance of these different modes, we focused only on the ten most significant pathways from the *pathVar* results obtained for the three cancer versus normal comparisons (Table S3.3 & S3.4). For all three cancers, the KEGG DNA replication pathway and REACTOME “DNA strand elongation term” had significant changes in both average and variability of expression. Other terms with changes in both average and variability were related to DNA damage response pathways, such as “base excision repair” (Table S3.3) for AML versus normal, and “non-homologous end-joining” for OVC versus normal (Table S3.4).

Five KEGG pathways had changes in variability only that were consistent in all three cancer comparisons; these were the pathways for Epstein-Bar virus infection, cell cycle, Fanconi anemia, lysosome and apoptosis (Table S2.3). The Epstein-Bar virus is associated with certain kinds of cancer like lymphoma or carcinoma. Apoptosis is also an important pathway for tumors because its inactivation is central in the development of cancer. Similarly, for the REACTOME terms, those unique to changes in variability were related to DNA repair and replication (SLBP dependent processing of replication-dependent histone pre-mRNAs) for the AML and GBM comparisons. For OVC, we saw several terms related to the cell cycle, e.g. G1 phase, cyclin D associated events in G1, cyclin A/B1 associated events during G2/M transition (Table S3.4).

Of the six two-group comparisons performed, it is interesting to note that the ESC and iPSC comparison also had the least number of significant pathways (Table S3.2) and this may have been due to the fact that all other comparisons were between a disease and normal group, or in the case of the mouse data, between two distinct regions of the brain. This result suggests that the degree of perturbation to a transcriptome in the presence of a tumor, or extra chromosome, or even a different anatomical region of the same organ, is greater globally, than how iPSCs differ from ESCs.

The observation that pathways were significant for changes in both average expression and gene expression variability reflects the different modes in which cells are using pathways to regulate transcriptional signals. For the cancer-based comparisons, it was interesting to see the common themes where pathways involved in DNA replication and DNA damage response had significant changes in average and variability (AML versus 1000 Genomes, OVC versus 1000 Genomes, Table S3.3). The reliance of DNA replication pathways may be to facilitate the proliferative nature of tumor cells, while the pathways that control DNA damage response are important for tumor cells to remain viable in the presence of increased rates of mutation. This result suggests that a critical factor to understanding how cancer subverts cellular pathways to promote growth and evade apoptosis more accurately may lie in focusing on how gene expression is being regulated based on average expression and expression variability from cell to cell, or from patient to patient.

In the one-group case, the variability measure that was the least correlated with the average expression was used. In the two-group case, the correlation between variability and average was also used to identify the most optimal variability measure; however, it became clear that this may not always be the only factor to consider. When comparing samples from the ovarian cancer cohort of the Cancer Genome Atlas (TCGA) with the Geuvadis/1000 Genomes dataset, nearly all genes were assigned to the low variability category based on the SD, whereas for MAD, the distribution counts were more evenly distributed across the three categories, making the discrimination of which pathways have differences in variability between the two populations a much easier task (Figure S3.1). In this example, the SD was the least correlated statistic with the average expression and the MAD was the second smallest, with the difference being negligible. Therefore, we draw the conclusion that using the correlation alone may not be sufficient in the two-group case, and it would also be constructive to consider the overall distribution of expression variability for all genes as another property in selecting an appropriate estimator of expression variability.

**Figure S3.1. The variability estimator can also produce different shapes of the count distribution, and facilitate the comparison between distributions.** Using the TCGA ovarian cancer (OVC) and the Geuvadis/1000 Genomes data sets, we noticed that the shape of the count distributions for the low, medium and high variability genes for SD and MAD were different. **A.** Based on the SD, the count distribution was (9,087, 9, 3) for the OVC data set, and (9102, 0, 0) for the 1000 Genomes data set. **B.** Based on the MAD, the count distribution was (5923, 1923, 1247) for the OVC data set, and (8190, 653, 259) for the 1000 Genomes data set. With the SD, the statistic places almost all genes into the low variability category, whereas using the MAD, genes are distributed with non-zero weight towards other variability categories, even though the distribution remains skewed towards lower levels of expression variability. Having a distribution with more non-zero weighting across the variability categories facilitates the comparison between the pathway and reference distributions.

**A.** SD.

**B.** MAD.
