# Supplementary material for: pathVar: a new method for pathway-based interpretation of gene expression variability
Source: PeerJ. 2017 May 23;5:e3334. doi: 10.7717/peerj.3334 (PMC5444375; doi:10.7717/peerj.3334)
Supplement: Text S4 [file peerj-05-3334-s005.docx]

**Text S4. Comparison of trends in variability between bulk versus single cell data.**

We contrasted the trends observed in expression variability using the gene expression data sets that profiled the human embryonic stem cells (ESCs). In Figure S4.1., the distributions of the expression variability densities highlight the differences between bulk and single cell data. Most genes have low variability in the bulk data, while for both sets of single cell data, we see more mass concentrated towards medium and higher levels of variability.

**Figure S4.1. Contrasting the expression variability distributions for bulk versus single cell ESC data for two populations of ESCs.** The densities show that bulk data is concentrated towards lower levels of variability, while both sets of single cell data have more genes with medium to high variability.

**
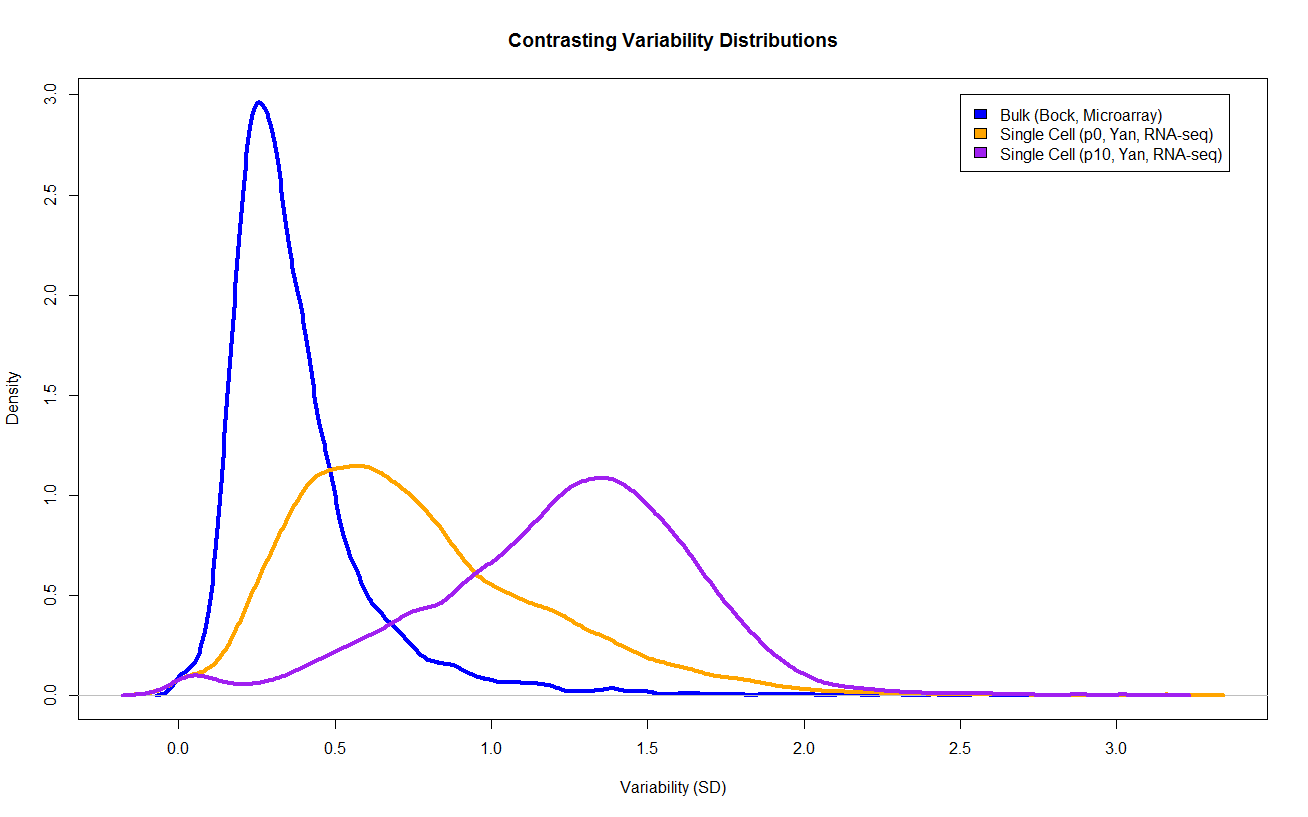
**

When we examine the relationship of expression variability with respect to average expression, we observed a much stronger dependency between these variables for the bulk data than the two sets of single cell data. This may support the suggestion that variability in single cells reflects a stronger biological origin than bulk data, which may represent sources of technical variation.

**Figure S4.2. The dependency between expression variability and average expression is stronger for single cell data compared to bulk data.** The lowess curves capture the overall trend between the expression variability and average expression. For single cell data, these curves demonstrate a more discernible relationship between average and variability, while for bulk data, the lowess curve indicates these variables are more independent from each other.

**
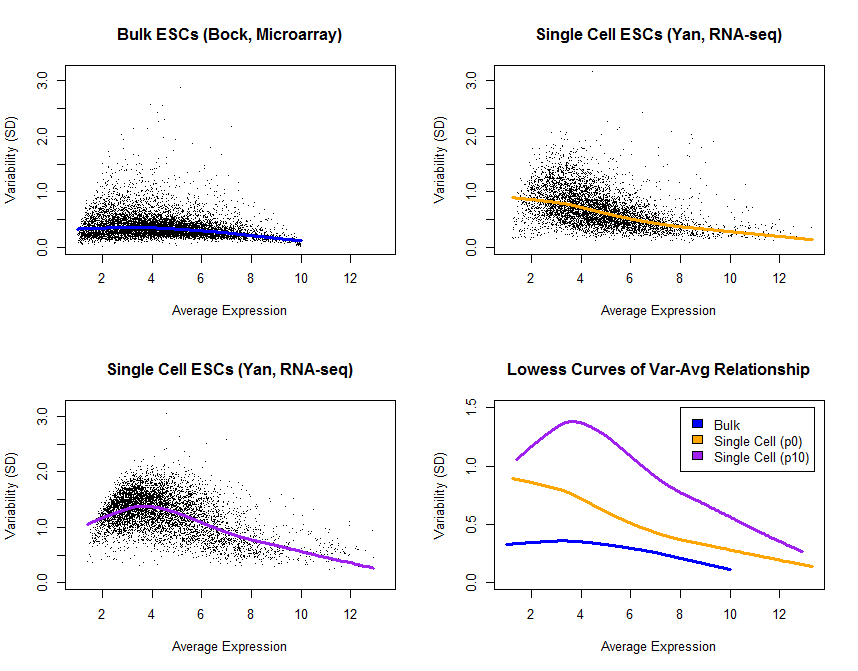
**
